# Simultaneous imaging of multi-pore sweat dynamics and evaporation rate measurement using wind tunnel ventilated capsule with infrared window

**DOI:** 10.1016/j.isci.2024.110304

**Published:** 2024-06-17

**Authors:** Ankush K. Jaiswal, Cibin T. Jose, Rajesh Ramesh, Vinay K. Nanani, Kambiz Sadeghi, Ankit Joshi, Krishna Kompally, Gokul Pathikonda, Heather N. Emady, Bhaumik Bheda, Stavros A. Kavouras, Konrad Rykaczewski

**Affiliations:** 1 School for Engineering of Matter, Transport and Energy, Arizona State University, Tempe, AZ, USA; 2 Julie Ann Wrigley Global Futures Laboratory, Arizona State University, Tempe, AZ 85287, USA; 3 College of Health Solutions, Arizona State University, Phoenix, AZ 85004, USA

**Keywords:** biophysics, devices

## Abstract

Sweat evaporation is critical to human thermoregulation, but current understanding of the process on 20 μm to 2 cm scale is limited. To this end, we introduce a wind-tunnel-shaped ventilated capsule with an infrared window for simultaneous infrared sweat imaging and evaporation rate measurement. Implementing the capsule in pilot human subject tests suggests that the common assumption of sweat being an isothermal film is only valid when the evaporation rate is low and sweat forms puddles on the skin. Before transitioning to this filmwise mode, sweating occurs in cyclic dropwise mode, displaying a 3x higher mass transfer coefficient in the same conditions. Imaging highlighted distinct phenomena occurring during and between these modes including out-of-duct evaporation, pulsating droplets, temporary and eventually lasting crevice filling, and individual drop-to-film spreading. In all, sweat evaporation is an impactful area that our results show is ripe for exploration, which can be achieved quantitatively using the introduced platform.

## Highlights

• Capsule with diffuser promotes computationally replicable parabolic flow over skin
• Imaging shows distinct sweating dynamics in the cyclic dropwise and filmwise modes
• The common isothermal sweat film assumption is valid only in limited conditions
• Mass transfer coefficient in cyclic dropwise mode is 3x higher than in filmwise mode

## Graphical abstract

**Figure:**
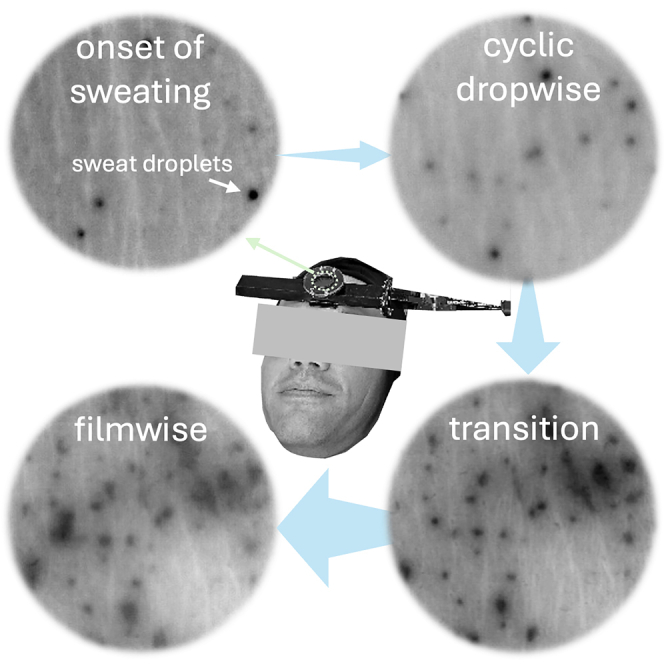

## Introduction

Considerable cooling off by sweat evaporation is a distinctively human feature. Having evolved about 10 times higher eccrine sweat gland density than other primates^1^^,^^2^ let human ancestors thrive in hot savannahs without overheating. Similarly, maximizing body,^3^^,^^4^ rather than surrounding,^5^ cooling achieved by evaporation of secreted sweat is critical for coping with increasingly frequent extreme heat events.^6^^,^^7^^,^^8^ Much like insight into underlying droplet and phase-change dynamics yielded improvements in industrial applications such as ink jet printing,^9^^,^^10^^,^^11^^,^^12^ a better quantitative understanding of the sweat evaporation mechanisms could yield ways to optimize body cooling achieved with the process (e.g., improved fabrics,^13^^,^^14^^,^^15^^,^^16^ actively ventilated clothing; ^17^^,^^18^ or recommendations for fan use during heat waves^19^^,^^20^). Such fundamental knowledge could also contribute to our basic understanding of wetness perception^21^^,^^22^ and benefit other applications, including the design of cosmetics^23^^,^^24^ and sweat sensors.^25^^,^^26^^,^^27^^,^^28^

While sweat production and secretion onto the skin has been characterized across length scales, from biochemical generation mechanisms to microfluidics of secretion and its rate variation at different skin regions,^2^^,^^21^^,^^29^^,^^30^^,^^31^^,^^32^^,^^33^^,^^34^ its evaporation has been approached almost exclusively from large skin regions or a whole-body perspective (i.e., microscale understanding of the process is lacking).^2^^,^^35^^,^^36^^,^^37^^,^^38^^,^^39^^,^^40^ Most common efforts to study the evaporation process from large skin regions use sweating instruments (from thermal manikin^41^^,^^42^ to torsos,^43^^,^^44^ plates,^45^^,^^46^ and even single "ducts"^23^^,^^47^) in the context of improving performance and safety apparel.^48^^,^^49^ The most anatomically realistic of the instruments, thermal manikins, dispense water onto a hard surface covered by a highly wicking and tightly fitting fabric (other approaches, including sintered porous metals and skin-like artificial sweating skins, exist^50^ but are not used to understand sweat evaporation).^51^ The wet-fabric approach mimics regional evaporation in the filmwise mode with fully wetted skin but cannot represent insensible or dropwise sweating modes or transitions between them (e.g., sweating onset or drying). From the theoretical side, sweat is most commonly treated as a thin isothermal film at underlying skin temperature.^38^^,^^39^ This film’s evaporation rate is modeled using the Lewis analogy that relates the convective heat transfer and mass transfer coefficients through a constant.^52^ While the isothermal thin film representation is helpful in apparel design and thermoregulation modeling, it cannot provide insight into finer mechanisms of sweat secretion and evaporation.

**Figure 1:**
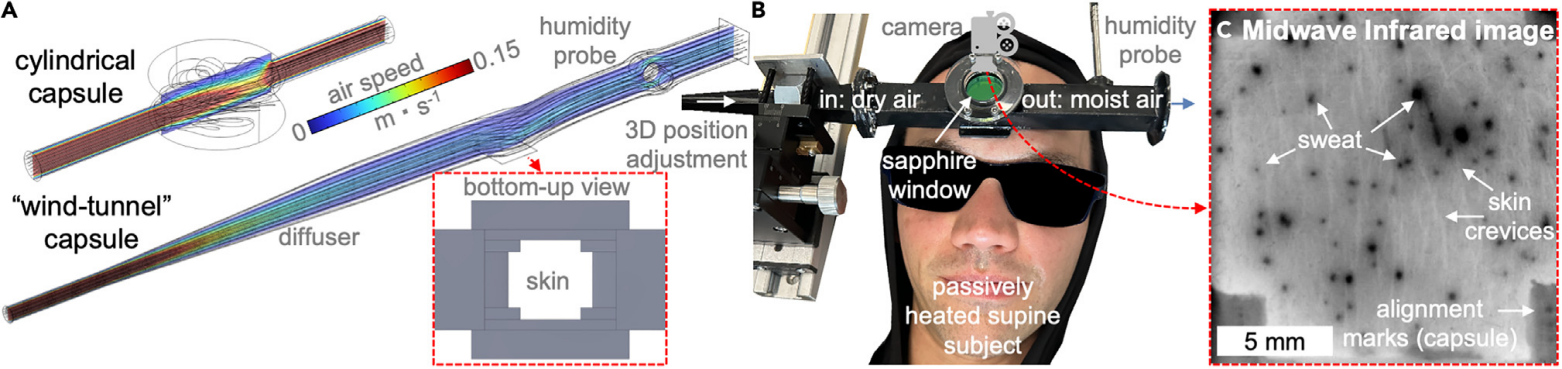
Overview of the wind tunnel ventilated capsule with infrared transparent window (A) Comparison of velocity distributions in a central vertical plane and streamlines within traditional cylindrical and the wind tunnel ventilated capsules at air flow rate of 0.5 L⋅min^−1^. (B) Annotated image showing the arrangement of the wind tunnel capsule for simultaneous measurement of the sweat evaporation rate and multi-pore scale imaging on a passively heated subject’s forehead. (C) An example of through-sapphire window mid-wave infrared image of sweat droplets evaporating from the skin surface.

To enable quantitative studies of the "multi-pore" scale (∼20 μm–2 cm) mechanisms of sweat evaporation, we synthesized methods employed by physiologists and engineers to create a platform for simultaneous sweat dynamics imaging and measurement of its evaporation rate in a realistic environment. In particular, we altered the geometry of ventilated capsules used by physiologists for over a century^2^ to measure sweat secretion rate based on wind tunnel design guidelines and enabled mid-wave infrared imaging (MWIR: 3–5 μm) of the skin through addition of a sapphire window. Figure 1A shows that traditional ventilated capsules are cylindrical^2^^,^^53^^,^^54^ (with one rectangular exception^55^), which induces highly swirling flow that facilitates evaporation of all sweat secreted onto the skin (i.e., the point of the physiological measurement). In contrast, flow simulations in Figure 1A show that the altered capsule with a diffuser and a rectangular flow section should promote a uniform flow across the skin that might be encountered in realistic situations such as walking or within a skin-clothing gap. In addition, the objective is for the flow within the capsule to be repeatable and readily computationally replicable to enable systematic interpretation of underlying physical mechanisms in *in vivo* observation.

Figure 1B shows that the sapphire window is above the exposed skin area and enables high-resolution MWIR thermographic imaging. Out of the numerous sweat visualization approaches that range from applying water-sensitive coatings on skin^2^ (or rolling films containing them across forehead^56^) to advanced two or three-dimensional microscopy,^57^^,^^58^^,^^59^^,^^60^^,^^61^^,^^62^^,^^63^^,^^64^ we selected MWIR thermography because it has several advantages demonstrated in prior imaging of mental sweating (i.e., rapid and short-lasting in response to non-thermal stimuli).^65^^,^^66^ The peak in the absorption of water at 3 μm makes the technique sensitive even to thin films of the liquid,^67^^,^^68^ while also providing fast imaging (10 Hz or more) over the relevant area (2 cm^2^) with resolution sufficient to capture even small sweat pores (∼20 μm). We validate that the altered capsule geometry yields uniform flow over the surface using particle image velocimetry (PIV) and benchmark the evaporation rate measurements using simple artificial surfaces, wherein liquid film of specified size is formed. Next, we implement the device in a pilot human trial, yielding illustrative evaporation rates and multi-pore scale sweat surface dynamics for the various stages of human perspiration. We use image analysis to quantify wet skin area fraction and relate it to the evaporation rate. We conclude by discussing the limitations of the ventilated capsule and imaging techniques highlighted by the pilot trial and approaches to mitigate these issues.

## Results

### The diffuser section creates uniform parabolic flow in the ventilated capsule

**Figure 2:**
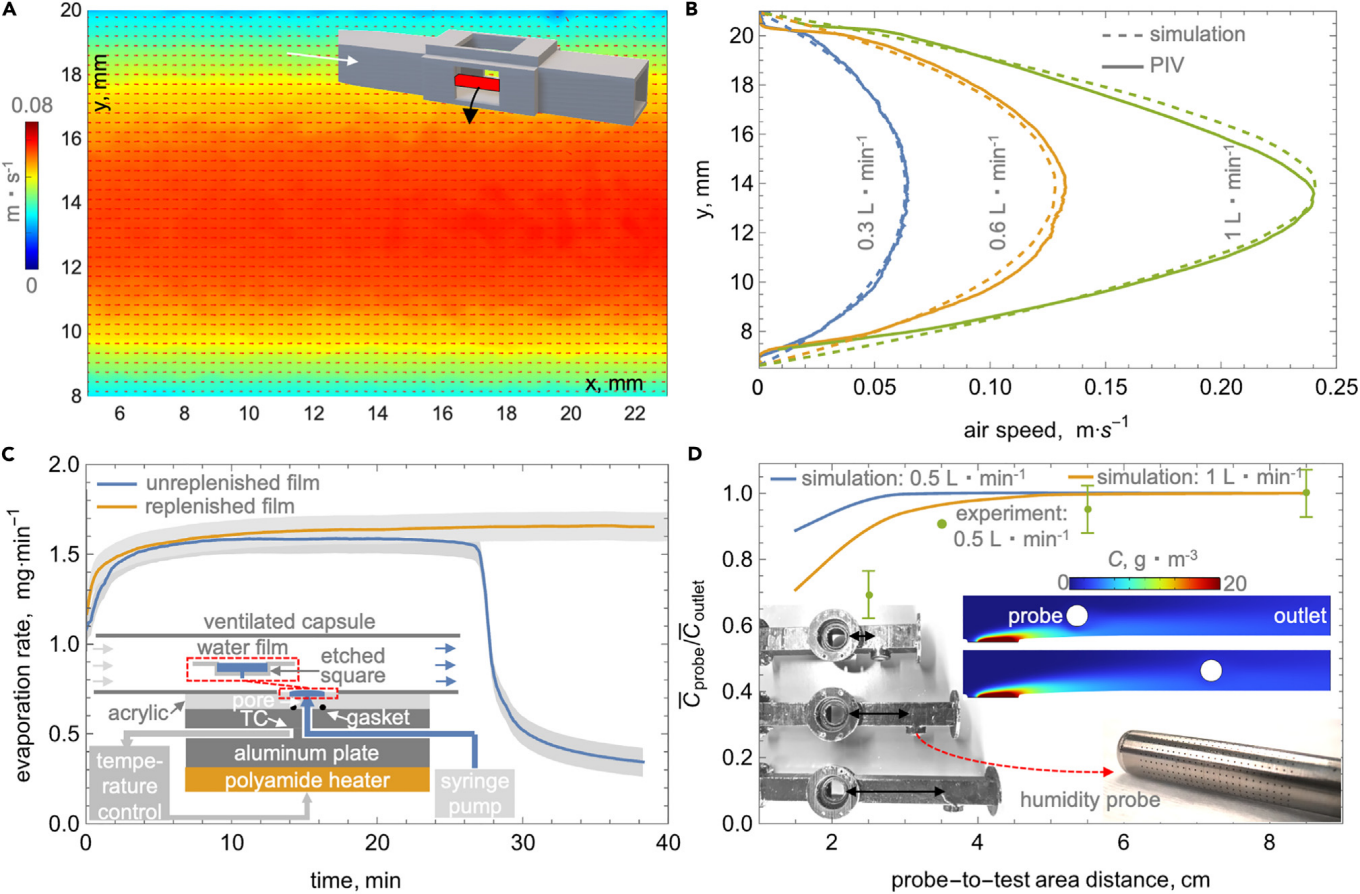
Characterization of air flow distribution within the wind tunnel capsule and benchmarking of evaporation rate measurements employing the device (A) Air velocity distribution on a central plane in a wind tunnel ventilated capsule with two acrylic windows with air flow rate of 0.3 L⋅min^−1^ visualized using particle image velocimetry (PIV). (B) Comparison of PIV-measured and simulated x-averaged [according to axis definitions in (A)] y-velocity profiles for air flow rates of 0.3, 0.6, and 1 L⋅min^−1^. (C) An example of experimental measurement of an artificial 0.64 cm^2^ square water film evaporation rate when the film is allowed to evaporate and when it is replenished to maintain equilibrium via a syringe pump; inset shows the schematic of the experimental setup. (D) Impact of humidity probe distance from evaporation area quantified using 0.64 cm^2^ square water film experiments and simulations at 0.5 and 1 L⋅min^−1^ air flow rate (to facilitate comparison, the ratio of average probe to average outlet water vapor concentrations is plotted); the insets show the evaporation sections of capsules with different probe locations (diffuser not attached), the perforated probe tip, and water vapor concentration profiles at 1 L⋅min^−1^ and two probe locations.

The diffuser section with a 3° expansion half angle recommended for wind tunnel design^69^ enables a smooth transition from the circular air supply tube to the rectangular evaporation section of the capsule and promotes the formation of the Hagen-Poiseuille flow. In particular, the PIV-visualized plane flow field in Figure 2A and velocity profiles in Figure 2B reveal the desired laminar parabolic flow,^70^ which is expected of laminar conditions, within the evaporation section across relevant air flow rates (up to 1 L⋅min^−1^ equivalent to mean speed of 9.25 cm⋅s^−1^). These experimental flow profiles are also readily replicated using simulations (see Figure 2B). In contrast, flow in a wide-angle diffuser with a half angle of 6° can reverse near the walls, while that in a sudden tube-to-rectangle expansion transition section (i.e., half angle of 90°) creates a high-velocity core and undesired stagnation regions near the walls (see the supplemental information—SI). Having demonstrated that the 3° half angle diffuser minimizes the flow separation and promotes the desired and readily computationally replicable Hagen-Poiseuille flow, we integrate the humidity probe into the capsule and benchmark the evaporation rate measurement.

### Optimization of capsule-humidity probe integration and evaporation rate validation using artificial sweating platform

We fabricated a simple heated square water film setup to systematically integrate the humidity probe into the ventilated capsule and benchmark the evaporation rate measurements (see Figure 2C and Methods Section for details). The evaporation rate from the film can be calculated as the product of the air flow rate ($\dot{Q}$) set by the digital flow controller and the difference in the inlet ($C_{in}$) and outlet water vapor concentrations ($C_{out}$):^70^

Equation (1) $${\dot{m}}_{e}=\dot{Q}\left(C_{out}-C_{in}\right)$$

Since we utilize ultrapure dry air, we can assume that $C_{in}=$ 0 and that ${\dot{m}}_{e}=\dot{Q}C_{out}$. Therefore, evaporation rate measurement only requires a single humidity probe after the exposed wet area. To facilitate the adoption of the wind tunnel capsule, we chose to use the cylindrical-tip humidity probe commonly employed in ventilated capsules in physiological studies (see inset in Figure 2D).^53^^,^^54^ Similarly, we use unit convention employed in physiology.^53^^,^^54^ Specifically, we report $\dot{Q}$ in L⋅min^−1^, $C_{out}$ in g⋅m^−3^, and ${\dot{m}}_{e}$ in mg⋅min^−1^ (or ${\dot{m}}_{e}^{\prime \prime }$ per cm^−1^ where relevant), which, due to the canceling of conversion factors, can be conveniently obtained by multiplying the flow rate and concentration in provided units.

To validate our evaporation rate measurements, we conducted two types of film evaporation experiments at $\dot{Q}$ of 0.5 L⋅min^−1^. First, we filled the etched square with water and let it evaporate without replenishing the liquid. Figure 2C shows that in this experiment, the evaporation rate exhibits a 20-min plateau at 1.6 mg⋅min^−1^ followed by a rapid decrease corresponding to the film dry-out phase. In the second experiment, we adopted the evaporation rate benchmarking approach employed previously in experiments on droplet evaporation.^68^ In particular, we continually supplied water to the square film at a rate of 1.6 mg⋅min^−1^ using a syringe pump. The plot in Figure 2C shows that when water was replenished at the same rate as it evaporated, the film dry out was avoided, validating our evaporation rate measurements. Next, we present the results of additional efforts to ensure the probe location does not impact these measurements under varied air flow rates.

The probe can provide an inaccurate measure of evaporated water if it is in a downstream location where the vapor has not diffused across the entire cross-section of the capsule. If the probe is located close to the evaporation area, the vapor concentration fields in the inset in Figure 2D show that only the bottom part of the cylinder is exposed to humid air. The plot in Figure 2D shows that such partial exposure can lead to 30% underprediction of the outlet water vapor concentration. We note that the underprediction of the experiments by simulations for the shortest probe-test area separation distance of 2.5 cm likely stems from simplifying the probe measurement area to an isotropic cylinder without specifically distributed perforations leading to the sensing element (see probe image in inset in Figure 2D). Both simulations and experiments performed using capsules with varied probe locations (see images in the inset) show that moving the probe 5 cm or more downstream of the evaporation area resolves the issue, even for the highest flow rate of 1 L⋅min^−1^. In that location, the entire probe surface is exposed to homogeneous water vapor concentration and measures values equal to those at the outlet (or measured with a probe located further downstream). Next, we describe the implementation of the wind tunnel ventilated capsule in pilot human trials.

### Pilot human trial: Simultaneous sweat evaporation rate measurement and MWIR multi-pore scale imaging

#### Optimized experimental setup and protocol: How to mount the wind tunnel ventilated capsule on a subject to enable high-quality imaging

To accommodate for the small depth of field of the MWIR camera, we minimized subject motion and disturbance in images by placing the person in a supine position, passive heating, and specialized capsule placement setup. As in many prior studies employing traditional ventilated capsules,^53^^,^^54^ we heated the subjects by passing water at 48°C through tubing integrated into a whole-body liquid perfused suit coupled with a constant temperature water bath (see methods). Through iterative protocol design with seven subjects with two measurement sites (forearm and forehead), we found the most consistent outcomes by placing the capsule on the subject’s forehead. We note that we exclude a "no heat strain" 34°C water perfusion period in related studies^54^ because, in a few cases, we observed sweat emerging from pores during this step. We also found that high-quality imaging required more rigid fixing of the capsule than obtained by gluing the capsules to the skin (as in prior studies). To achieve this, we fabricated a wooden platform with a rail that facilitates easy positioning of the capsule over the subject’s forehead. We achieved coarse leveling of the subject’s forehead by adjusting the level of the underlying head section of the bed. For finer position and rotation adjustments, the mounting of the capsule included two micrometers and rotational adjustment (see Figure 1B). We found that MWIR videos acquired using this method can be digitally stabilized and quantitatively analyzed through post-experimental image processing. Next, we describe example results of the capsule implementation, with single subject sweating progressing from the onset of cyclic dropwise to established filmwise modes.

#### From the onset of cyclic dropwise to established filmwise sweating with constant air flow rate

**Figure 3:**
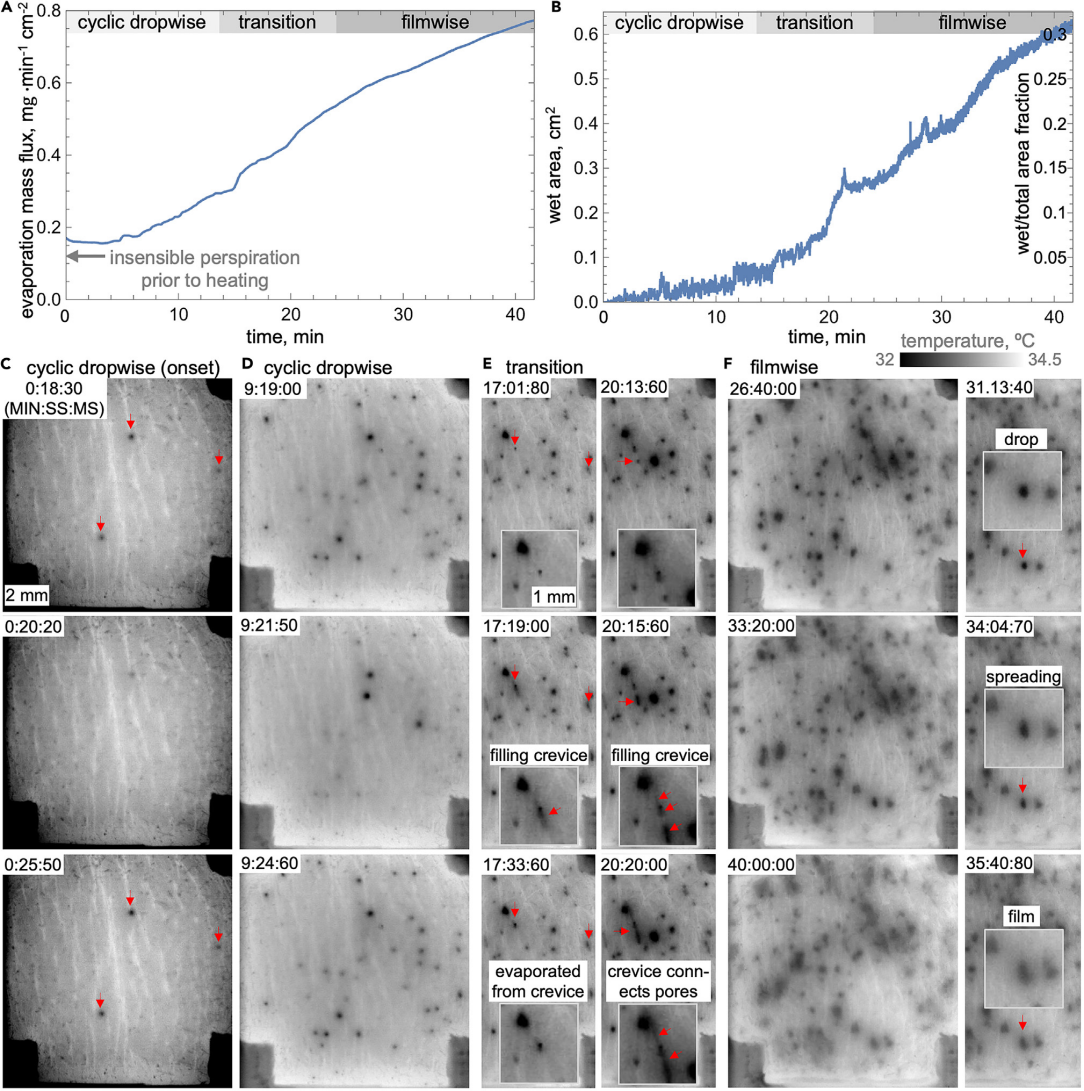
From the onset of cyclic dropwise to established filmwise sweating (A) The sweat evaporation mass flux measured with 0.1 L⋅min^−1^ air flow rate and per the 1.93 cm^2^ evaporation area and (B) corresponding absolute and relative wet surface area (i.e., covered by sweat) obtained from analyzing MWIR imaged surface sweat dynamics including (C) onset and (D) established cyclic dropwise sweating and (E) transition to (F) the filmwise mode.

**Figure 4:**
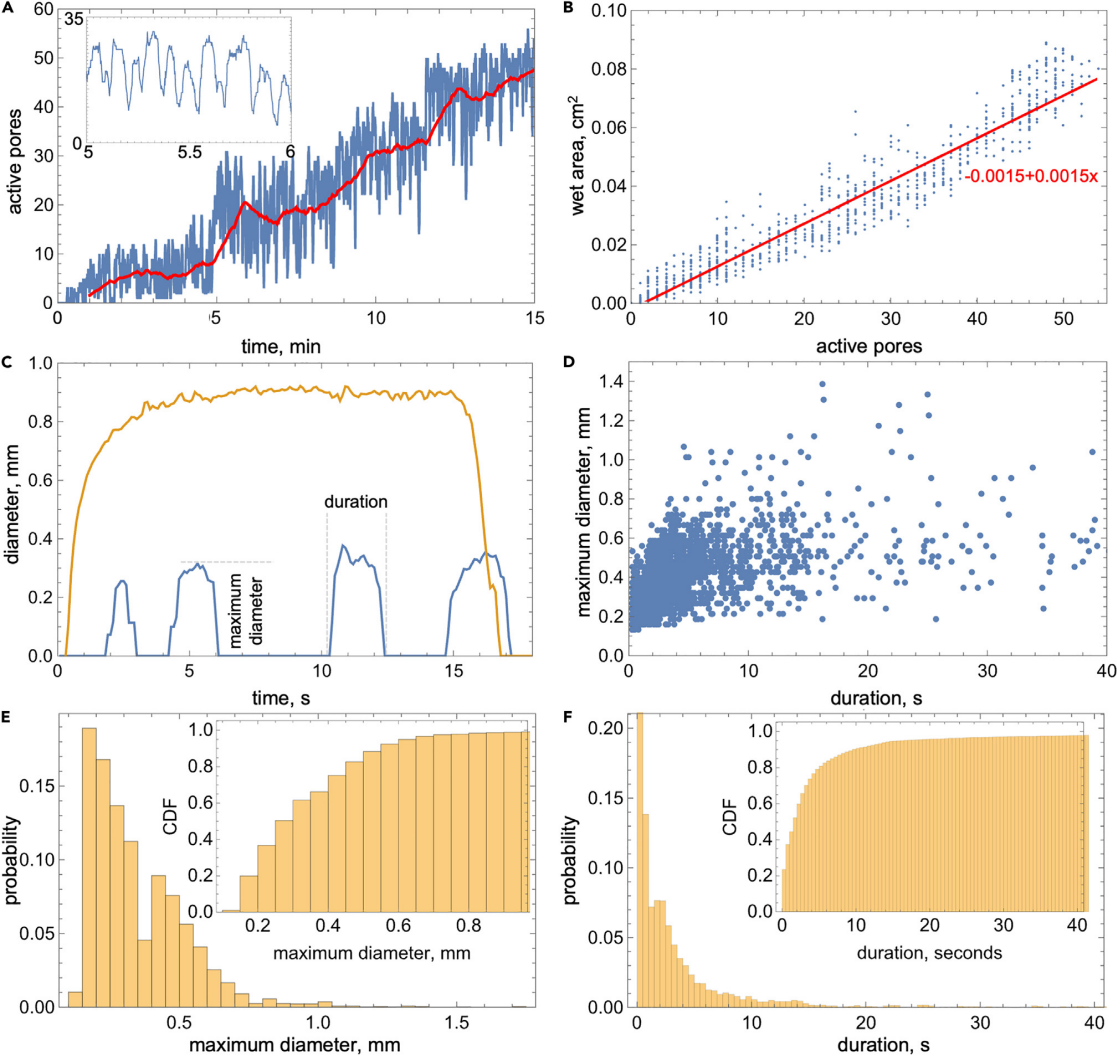
Droplet characteristics during cyclic dropwise sweating (A) The number of active pores (droplets) vs. time within the 1.93 cm^2^ evaporation area (the red line shows 1-min moving average). (B) The wet area vs. number of active pores (the red line shows a linear fit to the data whose equation is displayed). (C) Diameter vs. time plot showing examples of a long-lasting and large droplet (orange line) as well as multiple sequential (i.e., from same pore) brief and small droplets (blue line). (D) Maximum diameter vs. duration of the droplets. (E and F) Probability and cumulative distribution function (CDF) histograms of (E) the maximum diameter and (F) droplet duration.

Within 40 min of the MWIR detection of the first sweat droplet on the skin, the fraction of the wet skin increases to 0.3 while the evaporation mass flux increases from 0.15 to 0.8 mg⋅min^−1^cm^−2^ (see Figures 3A and 3B). The MWIR images in Figures 3C–3F and corresponding Videos S1, S2, S3, S4, and S5 show the emergence and transition between the cyclic dropwise and filmwise surface sweating modes, each displaying unique microscale dynamics. At the onset, sweat emerges onto the skin surface in droplet (circular) mode within distinct locations corresponding to sweat pores. The droplets are visible for a few seconds before the sweat evaporates and/or withdraws below the surface (i.e., it is no longer observable in the MWIR image). This process reoccurs periodically; therefore, we refer to it as cyclic dropwise sweating mode. While oscillating, the number of active pores, and thereby wetted surface area, progressively increases with time from just 1 to 3 at the onset to about 60 after 15 min (see Figure 4A). The wet surface area increases proportionally to the number of active pores, up to 0.1 cm^2^ or 0.05 (i.e., 5%) of the 1.93 cm^2^ of exposed skin area (see Figure 4B). According to a linear fit to these data, the wet area increases 0.145 mm^2^ for each droplet, equivalent to an average droplet having a 0.43 mm diameter. We observed about 3,500 individual droplets that, naturally, display a much wider range of diameters and durations. Figure 4C shows examples of droplets that briefly emerge from the same pore for 1–2 s and only spread about 0.1 mm beyond the pore (a maximum diameter of 0.2 mm). The figure also shows an example of a droplet that emerges from a different pore for 17 s and spreads far beyond its location of origin (a maximum diameter of 0.9 mm). Figure 4D shows that there is a trend for larger droplets lasting longer, while there are also many smaller droplets lasting more than 5 s. However, the histograms of the maximum droplet diameters and durations in Figures 4E and 4F show that short-lasting and small droplets occur most often. After about 15 min, new sweat surface dynamics begin to emerge, indicating the beginning of a transition away from the "pure" cyclic dropwise sweating mode hallmarked by the near-circular and non-interacting droplets.

Video S1. Onset of cyclic dropwise sweating (5x speed) related to Figure 1

Video S2. Cyclic dropwise sweating (5x speed) related to Figure 1

Video S3. Transition to filmwise: sweat spreading into skin crevices (5x speed) related to Figure 1

Video S4. Transition to filmwise: sweat bridges pores within crevices (5x speed) related to Figure 1

Video S5. Filmwise sweating (5x speed) related to Figure 1

The first feature of the shift away from pure dropwise sweating mode is the temporary filling of parts of crevices connected to active pores, which is followed by the formation of lasting (i.e., not oscillating) irregularly shaped sweat puddles and droplet-to-film transitions (see Figures 3E and 3F). In the MWIR images and movies, the filling of a crevice by sweat emerging from a pore is displayed as gradual and reversible darkening of up to 0.5 mm of the lighter-colored lines in contact with the droplet. Initially, this process is reversible due to the likely combination of sweat evaporation and retraction into the duct. However, as the transition progresses, the sweat spreads further within the crevices and can connect multiple pores. Another significant feature of the transition to filmwise mode is that many sweat droplets are present for longer duration, attain irregular shape, and have gradually decreasing MWIR contrast at the edges. This trend is highlighted in an example in Figure 3F of a near-circular dark droplet gradually spreading into irregularly shaped and substantially lighter puddle with difficult-to-define edges. Physically, these image contrast changes stem from sweat droplets with a shape likely resembling a partial spherical cap spreading into a shallow, film-like puddle. The top surface of the droplet is substantially cooler than the skin temperature (i.e., is darker in the MWIR image) due to evaporation and thermal resistance of the water droplet. In contrast, despite evaporating, a thin sweat film poses a smaller thermal resistance, and therefore, its surface has a temperature much closer to that of the nearby exposed skin.

The proliferation of the droplet-to-film spreading during the filmwise sweating stage translates into a gradual increase in the evaporation mass flux and an increase in the rate sweat covers the skin surface (see Figures 3A and 3B). However, the rate of surface wetting within the observation area was slow compared to nearly entirely sweat-flooded skin on the outside of the capsule. Considering the increasingly limited utility of the MWIR imaging of the established filmwise mode (i.e., poor contrast making quantitative evaluation challenging) and the subject’s comfort, we accelerated the flooding of the skin surface by removing the capsule for 5 min. Next, we describe experiments conducted to explore sweat dynamics during forced drying out of the film.

#### The drying out of filmwise sweating with stepwise increasing air flow rate

**Figure 5:**
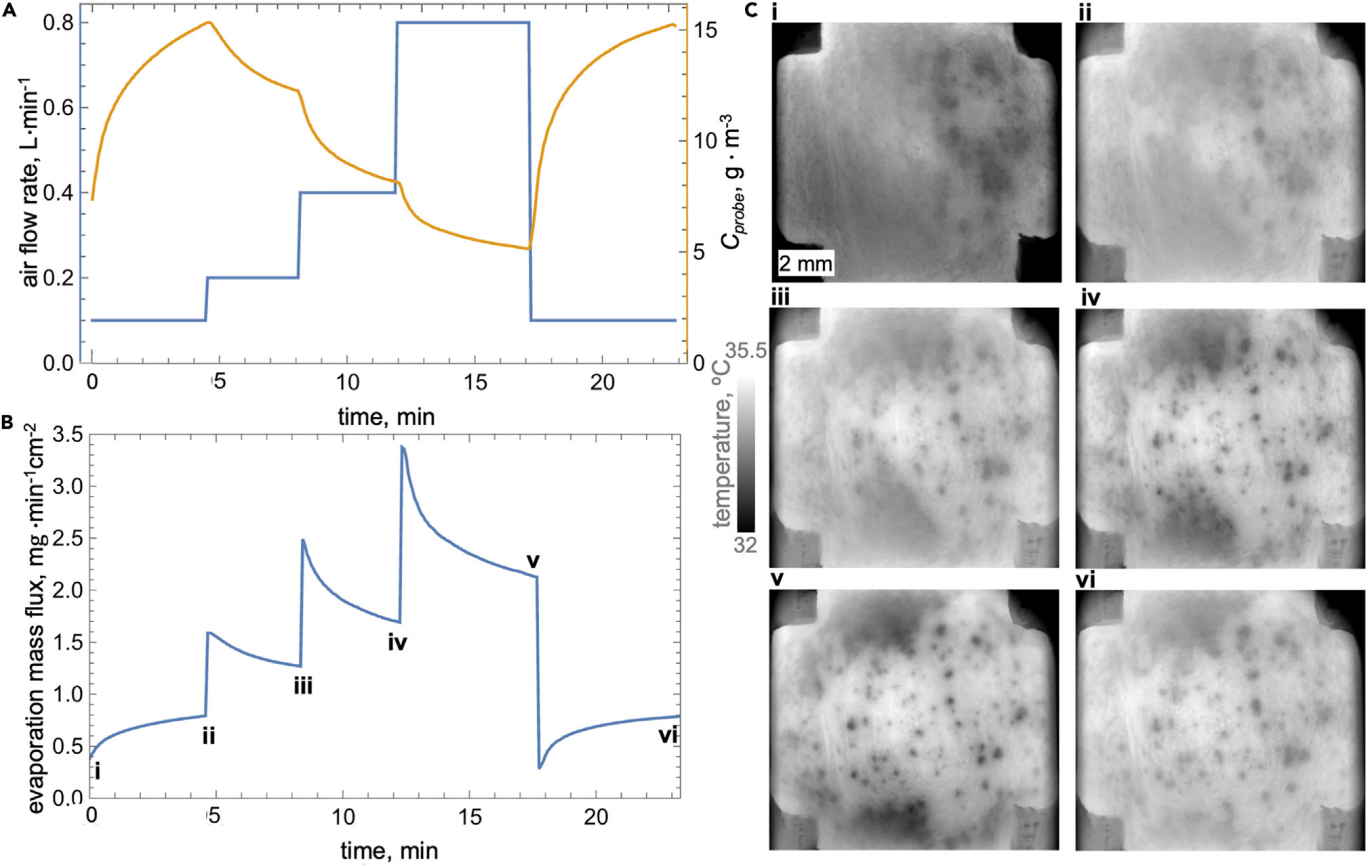
Forced drying out of filmwise sweating through increased air flow rate The time series of (A) the flow rate (blue line) and water vapor concentration (orange line) measured by the probe (C_probe_) for the 1.93 cm^2^ evaporation area and corresponding (B) evaporation mass flux and (C) six MWIR images corresponding to time points (i–vi) indicated in (B).

We reattached the wind tunnel capsule on the nearly entirely sweat-covered subject’s forehead to observe sweat evaporation dynamics during forced drying out of the filmwise mode. We doubled the air flow rate in three sequential steps. In particular, we set the air flow rate during the sequential steps to 0.1, 0.2, 0.4, and 0.8 L⋅min^−1^, with each step lasting 4 to 5 min. During the experiment, we determined the step duration *in situ* based on the observed rate of change in vapor concentration. The plots in Figures 5A and 5B show that after each step change in the air flow rate, the probe measured vapor concentration and evaporation mass flux decay in a near exponential fashion with an average fit-determined time constant of 1.5 min. This observation implies that after 4 to 5 min, the evaporation mass flux values were within 5%–10% of the quasi-steady-state value, confirming our decision of time-window selection during the experiment is appropriate.

The corresponding MWIR images in Figure 5C show that as the flow rate is increased, the initial near-isothermal sweat film progressively recedes, exposing a substantially warmer surface that is dotted by multiple cooler puddles corresponding to active pores (or pore ensembles) from which sweat is continually expelled. Many of these exposed puddles cyclically enlarge and decrease in size every few seconds, matching the oscillation period in the dropwise mode (see Video S6). After 5 min of the 0.8 L⋅min^−1^ step, we decreased the air flow rate to the initial 0.1 L⋅min^−1^. Within 5 min, the evaporation mass flux reaches the same value as at the end of the corresponding prior step (ii vs. vi), whereas the distinct sweat puddles diffuse into wider and lower contrast features (films) (Figures 5B and 5C). Next, we discuss our results in the context of prior physiological literature, highlighting the pluses of evaluating sweating using our method and its current limitations.

Video S6. Transition to filmwise: drop-to-film transition (20x speed) related to Figure 1

## Discussion

### The measured sweat evaporation rates and observed sweat dynamics match prior reports

The sweat evaporation rate and multi-pore scale surface dynamics quantified using the wind tunnel ventilated capsule closely match prior literature reports. Before heating of the subject, the skin surface is dry, and the evaporation mass flux is 0.11 mg⋅min^−1^cm^−2^, which is typical of imperceivable perspiration^2^^,^^30^^,^^54^ (average of values from numerous studies yields a forehead transepidermal water loss of 0.08 mg⋅min^−1^cm^−2^).^30^ After 25 min of heating, when the MWIR observable sweat secretion onto the skin begins, the evaporation mass flux increases slightly to 0.15 mg⋅min^−1^cm^−2^. Subsequently, the time series in Figure 3A shows that the evaporation mass flux increases to 0.8 mg⋅min^−1^cm^−2^ over 40 min, which closely matches measurements of Rutherford et al. despite some differences in the subject heating protocol.^54^ The time series in Figure 3B shows that during this time, the fraction of wet area quantified using MWIR images increases to 0.3, while the images visualize the sweating modes and related features.

The most prominent feature of dropwise sweating is its cyclic nature, which has been studied for at least 80 years^64^^,^^66^^,^^71^^,^^72^^,^^73^^,^^74^^,^^75^^,^^76^^,^^77^^,^^78^ While observed using various techniques, the reported cycle period matches ours of 1.5–13 s (average of 6.2 s), which can be directly correlated to the oscillatory sympathetic nerve activation of the sweat glands.^77^ As previously reported in the literature for thermal sweating on the forehead^74^^,^^78^ and other body parts,^73^ Figure 4A shows that the number of active sweat pores increases with time. The total number of active pores that we observed (up to ∼60 per 1.93 cm^2^, so ∼30 pore⋅cm^−2^) is lower than the ∼150 to 185 pore⋅cm^−2^ average distilled from numerous studies in the literature.^30^ However, for individual subjects, such numbers can vary drastically (e.g., 20–90 pore⋅cm^−2^ for the same experiment^74^). In addition, MWIR imaging is only helpful in detecting the active pores in the early stages of sweating and not during profuse sweating, which is often studied in physiology when more glands might be active (and, therefore, a sweat film forms). This is likely why MWIR imaging has been used predominantly to study mental sweating, which is too short for the transition from dropwise to filmwise mode.^65^^,^^66^ Accordingly, prior MWIR sweat imaging studies did not observe the temporary crevice filling, pore bridging through crevices, and droplet-to-film spreading features that occur during the sweating mode transition (Figure 3C).

The spreading of sweat from pores into the crevices and eventual pore bridging is likely facilitated by local stratum corneum hydration that causes water contact angle to decrease, facilitating sweat flow into the skin "trough." The stratum corneum is the epidermis’s outermost layer, and its near-complete hydration takes about 5 min of continuous contact with the liquid.^79^ In our experiments, the contact of the stratum corneum with sweat is cyclic, explaining why it took at least 15 min before we observed crevice filling and, therefore, the hydration process. The hydration process likely makes the surface more hydrophilic, facilitating the flow into the crevice through capillary forces.^80^ The contact of the stratum corneum with water droplets has been reported to cause a gradual decrease of the contact angle over a few minutes.^81^^,^^82^ Our wettability measurements were performed outside the capsule and only during major shifts in the experiments (e.g., taking the capsule off), but they do show that the contact angle decreased from about 60 to 70° (matching the typical contact angle of unwashed skin^82^) to below 10° (i.e., spreading into a film on sweaty skin) during the 40-min observation period. Consequently, the stratum-corneum-hydration-enhanced wettability seems like a plausible mechanism driving the crevice filling. However, a deeper investigation of the topic is warranted since the wettability of skin is highly complex and, depending on a myriad of factors, can range from highly hydrophobic to hydrophilic.^81^^,^^82^^,^^83^ Eventually, individual droplets begin to be connected through the sweat within the crevices, initiating gradual flooding of the surface beyond the pores.

Bridging of pores via crevices and drop-to-film spreading, not classical droplet coalescence, is the main mechanism underlying switching the sweating mode to filmwise. In particular, even in humid conditions, skin flooding through pinned droplet coalescence that drives dropwise-to-filmwise condensation transition is unlikely because the typical sweat pore separation distance is greater than the radius of most sweat droplets. Besides hands and feet, the forehead has one of the highest sweat pore densities on the body. The average 185 pore⋅cm^−2^ forehead pore density^30^ translates to about 0.7 mm separation distance on a hypothetical square grid distribution. The cumulative distribution histogram in Figure 4E shows that 95% of maximum droplet diameters are below 0.7 mm, making coalescence of two large droplets emerging from neighboring pores highly unlikely. Our observations show that instead of coalescing, most individual droplets on the surface spread into a film (see Figure 3F and Video S6). The 3- to 5-min period for this transition correlates with the time required for hydration of the stratum corneum, suggesting a decrease in skin wettability surrounding the droplet as a likely underlying mechanism. Therefore, the transition from dropwise to filmwise sweating and progressive covering of the skin with a patchy sweat film occurs through crevice-mediated pore bridging and drop-to-film transition, both likely driven by stratum corneum hydration. Collectively, these pore-level sweat features contribute to an increase in the rate of surface wetting between the two modes (see Figure 3B), but the impact of this process is only illuminated when it is directly compared against the measured sweat evaporation rate.

### Simultaneous sweat evaporation rate measurement and quantitative imaging reveal that the mass transfer coefficient in dropwise mode is 3x higher than in filmwise mode

**Figure 6:**
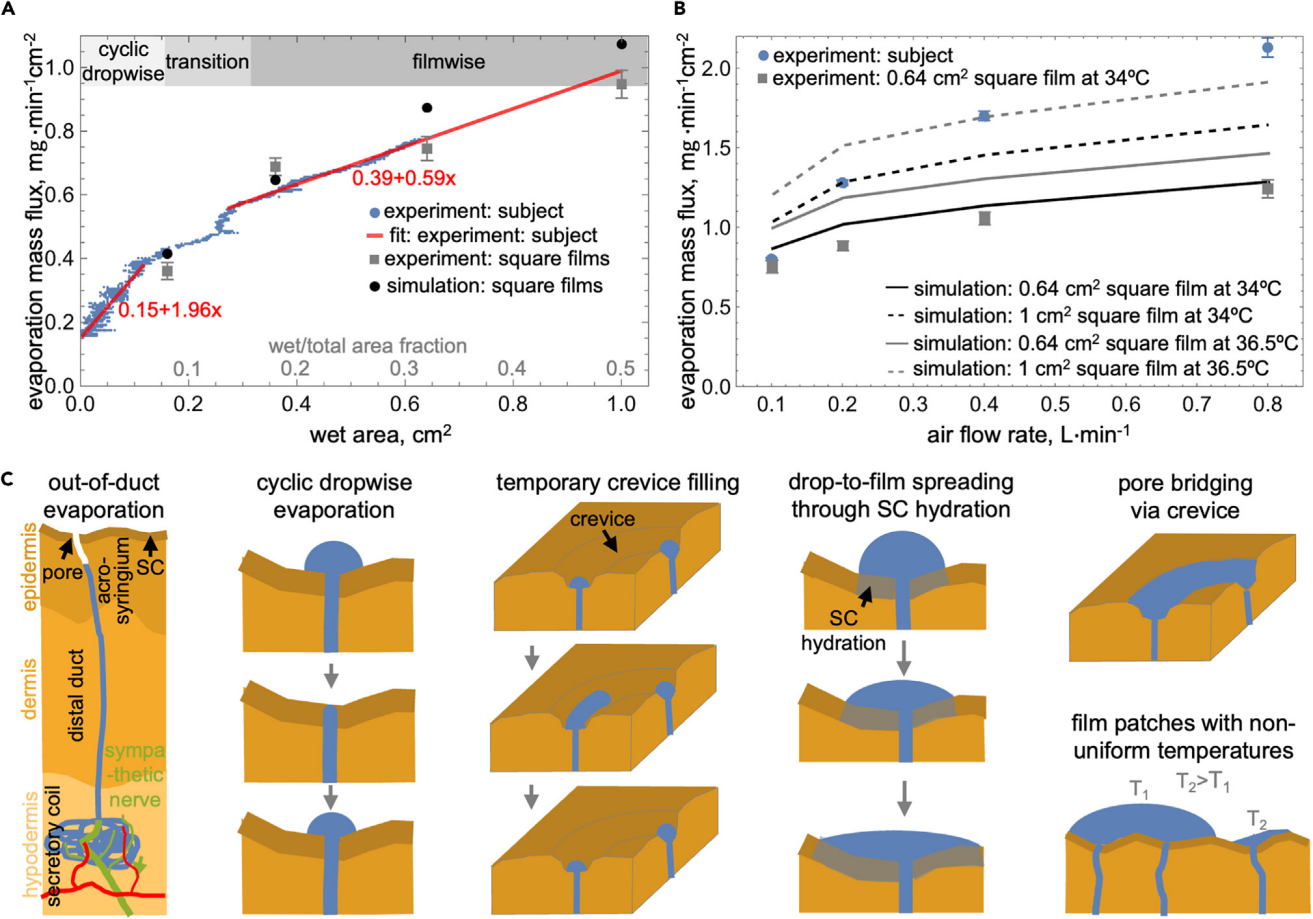
Synthesis of the evaporation rate measurement and MWIR image analysis (A) Evaporation mass flux vs. wet area (or wet area fraction) under constant air flow rate (0.1 L⋅min^−1^); results for simulated and measured evaporation from square water films with a varied area are also shown. (B) The forced "dry out" experiments: the quasi-steady state evaporation mass flux vs. air flow rate; simulations and measured evaporation of isothermal square water films at 34°C and 0.64 cm^2^ are also shown (additional simulation for 36.5°C and area of 1 cm^2^ are also included). (C) Schematic summary of the directly observed or implied pore or multi-pore scale processes underlying sweat evaporation including out-of-duct evaporation, cyclic dropwise evaporation, temporary crevice filling, drop-to-film spreading through stratum corneum (SC) hydration, pore bridging via crevice, and film puddles with non-uniform exterior temperature.

Employing the wind tunnel ventilated capsule, we can simultaneously quantify and relate to each other the sweat evaporation rate and multi-pore scale surface dynamics in realistic conditions that can be readily replicated computationally and experimentally with artificial sweating surfaces. Figure 6A synthesizes the time series in Figure 3 into a single plot relating the measured sweat evaporation mass flux and the wet area (or its fraction). In this perspective, the drastic difference in the regional slope makes the dropwise and filmwise modes immediately distinguishable. In particular, the rate of increase of the evaporation mass flux with wet area in the dropwise mode is more than three times higher than that in the filmwise mode. Remarkably, the simple square water film evaporation experiments and simulations varying the square area quantitively replicate the filmwise sweat evaporation results (see Figure 6A, the surface temperature in additional simulations and experiments was set to 34°C corresponding to the average observed in MWIR images of the filmwise mode).

The observed differences between dropwise and filmwise sweating modes can be quantitatively understood by considering the basic equation used to calculate the evaporation rate from an isothermal water or sweat film that covers a "wet" area ($A_{wet}$) that is only a fraction of the total area ($A_{total}$):^70^

Equation (2) $${\dot{m}}_{e}=A_{wet}h_{m}\left(C_{sat}\left(T_{sur}\right)-C_{\infty }\right)={\omega A}_{total}h_{m}\left(C_{sat}\left(T_{sur}\right)-C_{\infty }\right)$$

where ω= $A_{wet}/A_{total}$ is the wet surface area fraction, $h_{m}$ is the mass transfer coefficient, $C_{sat}\left(T_{sur}\right)$ is the saturation water vapor concentration at the exterior film surface temperature ($T_{sur}$), and $C_{\infty }$ is the water vapor concentration outside the mass boundary layer. Since for our experiments $C_{\infty }\approx$ 0, we can re-cast Equation 2 in terms of evaporation mass flux (${\dot{m}}_{e}^{\prime \prime }$):

Equation (3) $${{\dot{m}}_{e}^{\prime \prime }=\dot{m}}_{e}/A_{total}=\omega h_{m}C_{sat}\left(T_{sur}\right)$$

This leads to the derivative of the evaporation mass flux vs. wet surface area fraction (i.e., slope in Figure 6A) being:

Equation (4) $$\frac{d{\dot{m}}_{e}^{\prime \prime }}{d\omega }=h_{m}C_{sat}\left(T_{sur}\right)$$

The surface temperature, and therefore the $C_{sat}\left(T_{sur}\right)$, does not vary substantially between the two sweating modes. Consequently, the drastic difference between the slope for the two sweating modes stems from the tripling of the mass transfer coefficients between the filmwise and dropwise modes.

A large part of the 3-fold difference between the mass transfer coefficient in the dropwise and filmwise sweating modes is geometrical, but other more nuanced mechanisms are also likely to contribute. Sweat emerges from pores driven by a positive pressure gradient^34^ and has been observed on fingertips^60^ and assumed in simulations^64^ to have a spherical cap shape. Accordingly, a 2-fold increase in the mass transfer coefficient could be attributed to the geometrical difference between the surface of a circle imaged by MWIR and the hypothetical hemispherical shape of the sweat droplets.^64^ Naturally, the evaporation of a droplet that is replenished from beneath is highly complex^68^^,^^84^^,^^85^ and unlikely to result in a doubling of the evaporation rate compared to evaporation of a wet circle. However, even the idealized "geometrical" doubling of the mass transfer coefficient requires additional mechanisms to explain the 3-fold difference between the two sweating modes. The most likely explanation is that substantial evaporation occurs within the duct before the sweat emerges onto the skin^32^^,^^86^ and can be observed with the MWIR camera. Such "out-of-the duct" evaporation has been implied by large sweat loss of subjects exposed to arid conditions despite their skin remaining dry.^30^^,^^87^^,^^88^ In all, our pilot experiments indicate that sweat evaporation in the dropwise mode is substantially different than in the filmwise mode. As for the latter mode, the quantitative agreement of our *in vivo* results with simulations and experiments of evaporating square films implies that it can be approximated as the evaporation of a partial, isothermal, thin sweat film. However, substantially more complex trends emerge when the filmwise sweat evaporation rate increases under higher air flow rates.

### At high evaporation rates, the filmwise sweating mode is more complex than the isothermal thin film assumption

The filmwise evaporation mass flux increases with air flow rate despite the MWIR observable surface area decreasing substantially (see Figures 6B and 5C). In addition, the evaporation mass flux measured on the skin increases faster with air flow rate than simulations of isothermal thin square film and corresponding experiments would suggest (see Figure 6B). This trend holds even if the temperature of the simulated film is increased from 34°C to 36.5°C or its area is increased from 0.64 to 1 cm^2^. It is rather apparent in the MWIR imaging (see Figure 5C and Video S6) that filmwise sweating under a high evaporation rate is more complex than an isothermal film representation. Besides the re-appearance of pulsating sweat, albeit in puddles, not droplets, large temperature gradients emerge on the surface. The higher local surface temperature does not necessarily imply it is dry; sweat films as thin as 60 μm have been measured using optical coherence tomography (OCT).^57^ Instead, the large temperature gradients likely indicate that the thickness of the sweat films across different puddles is highly varied.

We can use thermal circuit analysis to support the assertion that non-uniform surface temperature at high evaporation flux implies non-uniform water film thickness. In particular, we can estimate that for evaporation mass flux of 2 mg⋅min^−1^cm^−2^, which with latent heat of sweat of 2,430 kJ⋅kg^4^^,^^70^ at 30°C is equivalent to an evaporative heat flux of about 800 W⋅m^−2^, a 60-μm and a 2-mm thick water film would result in 0.07°C and 2.7°C skin-to-exterior film surface temperature difference. In contrast, at the lower evaporation mass flux of 0.8 mg⋅min^−1^cm^−2^, which is equivalent to an evaporative heat flux of about 325 W⋅m^−2^, the temperature drop even across the 2-mm thick water film is only 1°C. These calculations explain why, at the lower evaporative mass fluxes observed at 0.1 L⋅min^−1^, the exterior temperature in filmwise mode is relatively uniform (e.g., see MWIR image for 40 min in Figure 3F). This characteristic leads to a uniform surface water vapor concentration and evaporation rate, which enables quantitative agreement of the *in vivo* measurements with simulated and experimentally measured evaporation from square water films. In contrast, such agreement between *in vivo* and simulated/experimental square film results breaks down when the evaporation rate is higher because the non-uniform thickness of water film puddles on the skin leads to a highly non-uniform exterior temperature, water vapor concentration, and evaporation rate. Therefore, even the filmwise sweat evaporation rate in many conditions does not agree with the commonly assumed isothermal thin film sweat representation.

In summary, to quantitatively study the sweat evaporation process on the 20 μm to 2 cm scale, we introduced a wind-tunnel-shaped ventilated capsule with a sapphire window that enables simultaneous evaporation rate measurement and multi-pore level imaging. The addition of the diffuser with a 3° half-angle provides parabolic flow within the rectangular section that is more realistic than the "cyclone" within traditional cylindrical capsules and could mimic real conditions (e.g., flow in-between clothing and skin or slow walking in calm wind conditions). We showed that this flow can be readily replicated computationally and over artificial sweating surfaces, facilitating quantitative interpretation of the *in vivo* experiments. For example, coupling of our experimental method with thermofluidic and bioheat transfer simulations developed by Drexelius et al.^64^ could be used to validate or advance modeling to provide accurate sweat droplet evaporation rate prediction solely based on infrared imaging.^64^ We demonstrated that the humidity probe integrated into the capsule provides accurate evaporation rate measurements across 0.1 to 1 L⋅min^−1^ inlet air flow rate using experiments with square water films and complementary multiphysics simulations. We also developed the experimental setup and protocols for implementing the wind tunnel ventilated capsule with MWIR imaging in human subject experiments.

The pilot human subject experiment employing the wind tunnel ventilated capsule suggests that the common representation of sweat as an isothermal thin film is only locally applicable in limited cases and revealed numerous features of multi-pore level sweating dynamics. In particular, our preliminary results suggest that the mass transfer coefficient for sweat evaporation in the cyclic dropwise mode is three times higher than in the filmwise mode. In addition, the agreement of square film simulations and experiments with *in vivo* results in Figure 6A shows that the latter mode is well approximated by an isothermal film only under low to moderate evaporative mass flux (e.g., 0.5–0.8 mg⋅min^−1^cm^−2^). Under more intense evaporation, the large heat flux likely leads to substantial differences in temperature drop across thin and thick water puddles and, therefore, a highly non-isothermal exterior surface (e.g., see bottom images in Figure 5C). Besides variations in temperature of the film puddles, the *in vivo* experiments also directly showed or implied several other multi-pore scale sweating features illustrated in Figure 6C, including out-of-duct evaporation and pulsating droplets in the cyclic mode and stratum-corneum-hydration-mediated crevice filling, pore bridging through crevice filling, and drop-to-film evolution in the transition to and in the filmwise mode. Although our results are preliminary, as we imaged only a single glabrous skin site on one subject, they suggest a plethora of intriguing micro- to macroscale liquid-phase phenomena occurring during sweating that significantly impact its evaporation. Considering how important it is to our thermoregulation in hot climates and other applications, sweat evaporation is an impactful area that is ripe for exploration, which can be achieved quantitatively using the introduced experimental platform.

### Limitations of the study

Implementing our method in the pilot human trial highlighted several limitations of the current ventilated capsule design and MWIR sweat imaging. In keeping the ventilated capsule design and experimental setup close to that used in physiological studies,^53^^,^^54^ we limited the lower end of evaporation rates that we can impose. In particular, by only having a single humidity probe at the capsule’s outlet, we are restricted to using "ultrapure" dry air ($C_{in}=$ 0). Consequently, even at the lowest air flow rate of 0.1 L⋅min^−1^, we measured a mass evaporation flux that would be, in average terms, in typical lab conditions (e.g., 24°C and 30% relative humidity) equivalent to the forehead being exposed to a high wind speed of 8.5 m⋅s^−1^ (estimated using Equation 2 with ω= 0.3, $T_{sur}=$ 34°C, ${\bar{h}}_{m}=0.00091{\bar{h}}_{t}$ via the Lewis analogy,^39^ where ${\bar{h}}_{t}=6.1V_{air}^{0.5}$ is the average heat transfer coefficient for the head and $V_{air}$ is the wind speed^39^). We note that the transfer coefficients are high because over the short length of the exposed skin segment the boundary layers are thin. If treated as flow over a 1.5 cm short plate (not over the entire head), the measured ${\bar{h}}_{t}$ would correspond to free stream velocity of only 0.3 m⋅s^−1^. While increasing the system cost, this issue can be easily resolved by adding an inline humidification component and a second humidity probe upstream of the diffuser. We also note that to be suitable for the limiting case of sweat evaporation without any air flow,^64^ the sapphire window would have to be heated to prevent fogging up of its internal surface. The humidity probe that we also adopted from physiological studies^53^^,^^54^ is highly accurate (±0.18 g⋅m^−3^) but has a response time of about 5–10 s that is too slow to detect evaporation rate oscillations due to cyclic sweat secretion.^75^ If these fine evaporation rate characteristics are of interest, another humidity probe must be utilized. Response time of the MWIR camera is fast (up to 60 Hz) and succeeds in imaging the pulsating sweat emerging from pores in the early sweating stages. However, we also observed several issues when employing this imaging technique to study sweat.

The most pronounced issues we observed with sweat imaging using MWIR are the small depth of field of the macro lens and difficulty interpreting images when temperature gradients on the skin are small. The small depth of field is particularly visible during *in vivo* experiments when, even with a relatively rigidly mounted capsule, slight movement of the subject can cause skin to "bow" and become unfocused within part of the viewing area. While our setup included a manual micrometer on the camera mounting, we found that continual focusing using the micrometer is challenging, a problem that might be addressed by an electronic micrometer. Even when the full image is in complete focus, small temperature gradients make quantitative image analysis challenging (e.g., it is difficult to judge where the edges of the water film patches in advanced filmwise sweating are in the bottom of Figure 3F or in Figure 3C). Quantitative MWIR of water droplets is also known to be challenging.^67^ In our pilot trials, we observed that large (∼1 mm diameter) sweat droplets with very high contrast appeared to cast a low-contrast "ring shadow" around them that disappeared when the droplets evaporated. This shadow could correspond to a physical process of interest, such as local thin film spreading, hydration of the stratum corneum, or conductive cooling of the skin around the droplet. However, it could also be caused by a fraction of the infrared radiation emitted from the skin within the ring area being absorbed by the side of the droplet.

While some progress in interpreting infrared images of sweat can be made with clever comparison of before and after sweat droplet appearance images^62^^,^^63^ or by interpreting using thermofluidic simulations the temperatures of droplet and its surrounding,^64^ it is difficult to implement during extended sweating when pores rarely become dry or when the surrounding might also be wet. A potential approach to addressing most of the issues with MWIR imaging is correlative imaging with another technique. In particular, optical coherence tomography (OCT)^57^^,^^58^^,^^59^^,^^60^^,^^61^ appears to be particularly well suited to this task, as it provides cross-sectional images of skin and its surface that would be ideal for aiding interpretation of MWIR images (e.g., droplet shape and film thicknesses). It is worth noting that other imaging techniques might be better suited for studying evaporation from single pores (diameter of 20–60 μm), as both MWIR and OCT in-plane spatial resolution are at best around 10 μm (OCT^58^) to 15 μm (MWIR). Lastly, we highlight that since this was only a pilot study demonstrating the implementation of the capsule and providing illustrative outcomes, results from the final subject (out of seven) with a fully optimized capsule and MWIR procedure are provided. We note that experiments with this subject were repeated three times over 10 days at the end of January of 2024, with comparable sweating mode and evaporation rate temporal evolution observed each time (the results with most optimized procedure from the last experiments were analyzed using image processing and are presented in the paper).

## STAR★Methods

### Key resources table

**Table:** 

REAGENT or RESOURCE	SOURCE	IDENTIFIER
**Deposited data**		

2024 MWIR 0.1 LPM example sweating dynamics in wind tunnel capsule”, Mendeley Data, V1	This paper	https://doi.org/10.17632/g6hb4r2mmc.1

**Software and algorithms**		

FIJI/ImageJ2.14.0/1.54f	ImageJ	https://fiji.sc
Comsol Multiphysics 6.1	Comsol Multiphysics	https://www.comsol.com

### Resource availability

#### Lead contact

Further information and requests for resources and reagents should be directed to and will be fulfilled by the lead contact, Konrad Rykaczewski (konradr@asu.edu)

#### Materials availability

This study did not generate new materials.

#### Data and code availability

• Data

The stabilized grayscale MWIR video and corresponding binary video resulting from image processing of the infrared data of the entire 45 min sweating process at 0.1 L⋅min^−1^ have been deposited on Mendeley Data and are publicly available. DOIs are listed in the key resources table.

• Code

This paper does not report original code.

• Additional Information

Any additional information required to reanalyze the data reported in this paper is available from the lead contact upon request.

### Experimental model and study participant details

The Arizona State University Institutional Review Board approved the human subject experiments. Since this was only a pilot study demonstrating the implementation of the capsule and providing illustrative outcomes, results from the final subject (out of seven) with a fully optimized capsule and MWIR procedure are provided. The subject was a 40 years old Caucasian male of eastern European ancestry, had a height of 175 cm, and weighed 69 kg. We note that experiments with this subject were repeated three times over 10 days at the end of January of 2024, with comparable sweating mode and evaporation rate temporal evolution observed each time (the results with most optimized procedure from the last experiments were analyzed using image processing and are presented in the paper).

### Method details

#### Capsule fabrication and sensor integration

The digitally designed diffuser and evaporation sections of the capsule were 3D printed (Prusa i3 MK3S with 0.1 mm 'detail' setting, 30% infill) using polyethylene terephthalate glycol (PETG, Overture 1.75 mm) filament. All printed parts were coated three times with epoxy (XTC 3D) to seal any potential gaps. To facilitate conformal coating, the epoxy was diluted with acetone before each application. After curing the coating for 24 h at 22°C to 24°C, excess parts blocking, for example, the flanges were manually filed down. Custom gaskets (square or circular) for the flange joining the capsule sections, the 2.54 cm diameter and 0.5 cm thick sapphire window (Thorlabs WG31050-E1 with anti-reflective coating), and interface with the acrylic artificial sweating surfaces were made using splicing kit (McMaster Carr 9410K11). The diffuser section and evaporation section were connected using a flange that has eight holes with a diameter of 2.6 mm and are fastened together with M2 screws (McMaster-Carr 90116A020) and nuts (McMaster-Carr 94150A305). One of the flanges has groove for an O-ring (cross section of 2.5 mm with internal diameter of 21.95 mm). Another flange with four holes was printed on the outlet of the evaporation section for a cap that is used during outlet air leak test. In the test, the capsule is fully closed (see image of the cap in Figure S2) and pressurized to about 8000 Pa, as measured by an inline pressure gauge. The cap was laser cut to a diameter of 39 mm, had a laser etched groove for an O-ring, and was secured to the capsule’s flange using has four set of screws. Indicating good sealing capability, the capsule can hold the pressure for over 10 min.

The high accuracy (±0.8% relative humidity or ±0.18 g⋅m^−3^) compact humidity probe (Vaisala HMP9) with digital transmitter (Vaisala Indigo202, powered through wall plugged power adapter) was inserted into a 7.9 mm hole on the side of the evaporation section using a grommet (Buna-N, MS 35489-91). We calibrated the probe using saturated aqueous salt solution in a closed container.^89^ We placed the probe through a hole with a grommet in an airtight plastic storage container with a 29.5 mL capacity along with the salt solution. We made the saturated solution with distilled water and NaCl (ACS reagent, ≥99.0%, Sigma Aldrich). After closing, we allowed the system to reach equilibrium over 24 h. The standard reference point is 75.5 ± 0.2% relative humidity (RH) at 20°C, however, if temperature cannot be maintained at ±0.1°C, drift of the humidity reading exceeding 0.5% is expected. We did not control lab temperature and had drift up to 1.2°C, resulting in a larger uncertainty (75.4 ± 2.8% with temperature of 22.3 ± 0.6°C (±1 standard deviation).

The capsule was perfused with Ultra Zero Air (Airgas, less than 2 ppm of water) from the high-pressure cylinder. The pressure from the cylinder drives the air flow that was regulated using a digital mass flow controller (Alicat Scientific, MC-1SLP-D-DB9M/5m, standard accuracy of ±0.1% of full scale or ±0.6% of the reading, powered through wall plugged power adapter). We fabricated over 10 of the wind tunnel ventilated capsule devices over two years of the project and did not observe any device-to-device differences in heated square water film platform experiments. We note that to assess the 3D printed and coated part compatibility with moist environments, we tested the rate of water absorption by the device. In particular, we 3D printed and coated with epoxy two samples immersed in water for 24 h. The mass of the samples before and after the 24 h immersion as shown in Table S1, demonstrating a negligible water absorption level (∼1% or lower).

We note that very high air flow rate could locally decrease skin temperature through evaporation. In general, sweat secretion rate is modulated by core, mean skin, and local skin temperature. However, the agreement between sweat secretion rate measurements done using traditional high flow rate ventilated capsules and absorbent pads implies that any local temperature decrease within capsule has negligible impact on the secretion rate.^90^ The Figure S1, Table S1, and Figure S2 in the SI show related details of the capsule geometry, humidity probe calibration procedure, ventilated capsule water absorption (below 1% in 24 h of soaking), and air pressure sealing tests.

#### Heated square film platform

We developed a simple platform for dispensing square 0.5 mm thin heated water films at highly controlled flow rates to optimize the capsule design and benchmark its evaporation rate measurement. The setup consists of four layered acrylic plates that house a 3 cm by 4 cm by 0.5 cm aluminum block whose temperature is set using two thin film polyimide heaters (Icstation Polyimide Heating Elements Film, 5V, 1W, Stripboard Mat) with feedback provided by an embedded T-type thermocouple. The heater is controlled using on/off basis with a benchtop controller (Omega CSi8D) with AC-to-DC converter attached to the power output (5 V DC). The metal block has a horizontal channel (2 mm inner diameter) for the supply and preheating of the water that is connected to a vertical channel that dispenses the liquid onto the top surface of the block. The flow of water is set using a syringe pump (NE-300 Just Infusion Syringe Pump) with a plastic dispensing needle (McMaster Carr 75115A671) with a luer lock connection to the syringe pump side being embedded into the 2 mm internal diameter hole in the aluminum block. The water "reservoir" on the metal surface is created using an o-ring (Silicone 70 durometer 568-011) that is pressed against the metal with a 3.1 mm thick acrylic plate. The latter has patterns etched on both sides using a laser cutter (Full Spectrum Laser PS20), with a gasket groove on the bottom and 0.5 mm deep upper square with an area varying between 0.16 and 1 cm^2^. The water reservoir is connected to the bottom of the etched square through a 0.1 mm laser-cut hole. Laser-etching produces a micro-textured and hydrophilic surface that facilitates film formation within the square. To conduct experiments with external film temperature at about 34°C, the heater set point was adjusted for each flow rate based on input from MWIR images. Images of the setup are shown in Figure S3.

#### Coupled multiphysics simulations

The multiphysics simulations were performed using Comsol Multiphysics 6.1 and coupled Laminar Flow, Heat Transfer in Moist Air, and Dilute Species Mass Transport physics. Only the internal air domain was simulated with inlet conditions being a fully developed laminar flow within the round supply tube at a typical temperature of the lab (23°C) and water vapor concentration approximated as 0 g⋅m^−3^. For all physics, the outlet of the capsule was set to "Open Boundary" condition. The exposed "skin" area of 15 by 15 mm was treated as isothermal at 34°C with a centrally placed square (0.16–1 cm^2^) having "Wet" boundary condition in the mass transport physics (i.e., water vapor concentration is equal to saturation concentration at the surface temperature). The mesh refinement study and images of the utilized mesh are shown in Figure S4, while further details of the model formulation are described elsewhere.^5^

#### PIV measurements

The planar PIV was performed using a New Wave Nd:YAG laser with an output power of 130 mj/Pulse and a 11-megapixel TSI PowerView CCD camera. A series of optics were used to redirect the laser, passed through a cylindrical lens to make a thin sheet about 1 mm wide, and passed through the nozzle to illuminate the streamwise-spanwise plane, as shown in Figure 2A. The laser emits 532 nm light and was used to illuminate the seeding particles in the camera’s field of view. A Laskin nozzle filled with olive oil provided the necessary seeding particles for these experiments. A calibration target was used to convert the images from the camera into real-world coordinates to obtain the velocity vectors. The data was processed on LaVision Davis 11 using multi-pass grid refinement to the final interrogation window of 32 pixel × 32 pixel (with a 50% overlap). This gives a final vector spacing of 0.08 mm⋅vector^−1^ in x- and y-directions.

#### Experimental setup for human trials

An experiment setup for human trials consists of the ventilated capsule, constant temperature bath (VWR AP28R-30-V11B), water tube perfused full body heating suit with a hood (Compcooler Full Body Cooling Garment with stretchable fabric XS/S and M/L), custom machined MWIR camera mount, adjustable reclining chair for subject (Paddie Electric Height Adjustable Bed Chair, Electric Lift Massage Table 3-Section Folding), heated gloves, and additional heated blankets (Homlyns, 127 by 152 cm with 120V). A capacitive skin hydration meter (Delfin MoistureMeter SC) was used to measure skin hydration outside of the capsule throughout the experiments, with initial values of about 30 (no unit) increasing to over 100 by the end of the 40 min experiment. A Sony A7Siii camera with Venus Optics Laowa 24mm f/14 Probe Lens is used to image representative water droplets (∼1–5 μL) of the subject’s skin near the capsule. The water contact angles before heating varied between 60 and 75° and were below 10° as the capsule was taken off between experiments (measured nearby but not on the imaging site).

The capsule mount consists of a gliding rail (Firgelli, 30 cm) attached to a wooden platform that is itself secured using a robust camera arm (AmScope Articulating Stand with Clamp and Focusing Rack for Stereo Microscopes) to a nearby table and allows for rapid and significant movement of the capsule (i.e., moving in and out of the site area). The capsule mounting on the rail has an assembly of two manual micrometers (Newport with 2 cm travel) that can be adjusted with high precision without moving the subject. The capsule diffuser itself was manually screwed into a 17.2 mm inner diameter machine screw nut (which also provided a ∼30° rotational adjustment) that was secured to the micrometer using a thin aluminum plate and four screws.

The full body heating suit has many tubes distributed across the entire surface area for uniform heating. As in related studies,^53^^,^^54^ water at 48°C supplied through the constant temperature bath is circulated in the suit. The values of temperatures and flow rates of water entering and exiting the suit are also monitored with the help of a rotameter and T-type thermocouples. The experiments are performed in a supine posture, with the major adjustment of the human subject position with respect to the MWIR camera carried out by adjusting the chair. An additional micrometer was added to the camera mount to adjust focus without touching the lens. A heated blanket is placed on the chair to minimize heat losses from the circulated water to the surroundings. Subsequently, a 76.2 cm wide waterproof exam paper (TOA Disposable Polypaper waterproof exam paper) is used to cover the blanket and prevent its saturation with the subject’s sweat. Afterward, the subject wearing the heated suit was asked to lie on the chair. The subject was covered with another layer of the exam paper and a second heated blanket. In addition, the subject also wore a pair of heated gloves to cover the hands. The capsule’s contact with the skin was sealed using double-sided skin tape (skin-compatible double-sided tape, BearKig) placed onto the capsule surrounding the skin opening. Images of the experimental setup are shown in Figure S7.

#### Experimental protocol for the pilot human trial

The Arizona State University Institutional Review Board approved the human subject experiment. All participants were screened using inclusion criteria (between 18 and 55 years old with no history of significant health issues such as high blood pressure or current symptoms that mild hyperthermia might exacerbate) and provided written informed consent before participating. The subjects were asked to avoid alcohol the night prior to experiments and have a caffeine-free light breakfast and at least 0.5 L of water 2 h before the experiment.

As a first step upon arrival, the subjects were asked to weigh themselves without clothing, a process that was repeated after the experiment to determine total sweat loss. Wearing T-shirt and shorts, the subjects put on the full body suit. The capsule was purged before every experiment to remove any absorbed moisture due to ambient air exposure. Before laying down on the chair, the subjects were instrumented with an external core temperature sensor (CORE sensor). Throughout the experiment, the core temperature increase was typically 0.5°C–0.8°C, as expected from our heating procedure.^54^ After the subject lay down, the capsule was mounted on their forehead while heating with 48°C water was started. Sweating was typically detectable with the MWIR camera and visually after about 20–25 min of heating. The subject comfort level was verbally assessed throughout the experiment, which was terminated early if the subject was uncomfortable. When requested, the subject was given a weighed amount of water throughout the experiment. After the experiment, the subjects were asked to weigh themselves, rest, and drink water for 15 min. About 0.75 to 1 kg net water loss was indicated through weight change and accounting for water intake.

#### MWIR imaging and image analysis

The MWIR videos were captured using an FLIR MWIR 6701 camera with a 50 mm f2.5 macro lens. The instrument has a detector with 3–5 μm spectral range and 640 × 512 pixel count. The images were recorded at 10 Hz (camera range is 0.0015 Hz–60 Hz), double the acquisition rates used in prior MWIR studies on mental sweating.^66^ The lens zoom was extended to provide an image of the exposed skin area within the ventilated capsule, translating to 26.7 μm per pixel spatial resolution. The movies were exported from the FLIR Research Studio software (Teledyne FLIR) in.avi format and grayscale and imported into FIJI/ImageJ 2.14.0.^91^ Once the temperature scale was cropped, the unavoidable shaking in the recording related to the subject’s breathing was removed using the Image Stabilizer plugin with default settings. The over 25,000 images covered over 40 min of sweating in different modes and were converted to binary (wet/dry) using Auto Local Thresholding. However, the details of the process required adjustment to the different modes of sweating. In particular, the iterative trial demonstrated that best outcomes were achieved for established cyclic dropwise sweating using the Phansalkar auto local threshold with radius varying from 15 to 25 pixels, while in the filmwise mode, the mid-gray auto local threshold with a radius of 35 pixels was preferred. Post thresholding, several additional processing steps on the binary images were performed to minimize noise and smooth the droplet shapes, including "binary/close", "binary/dilate", and “filter/Gaussian blur” with 2 pixel radius (the latter was followed by another single-value based thresholding step to re-convert to binary). We note, however, that even blending of the approaches using "image calculator" often yielded imperfect results, as visually judged by comparison of the original and processed videos. Consequently, multiple manual edits were also conducted to eliminate major artifacts.

The total area and individual droplet areas within each binary image was determined using the “analyze/analyze particles” function. To determine the maximum diameter and duration of each droplet across multiple sequential images, the image stacks with varying time were analyzed using the “analyze/3D object counter” function (with time treated as the “z axis”). To remove noise, “droplets” that only lasted two slices were filtered out. While this substantially reduced the total number of analyzed droplets (down to 3,500), it had negligible impact on the total area within each slice (analyzed using the “analyzed particles” function in post-3D filtered stack).

### Quantification and statistical analysis

All experiments employing the heated square film platform were repeated three times. The random error for the evaporation rate was calculated using 2-sided T-student distribution with 95% confidence interval based on standard deviation of the experimentally measured, $\sigma_{\dot{m}}$, as $U_{random}=4.3\sigma_{\dot{m}}$. The systematic error based on measurement uncertainties was calculated from error propagation of Equation 1 with $C_{in}=$ 0 (i.e., ${\dot{m}}_{e}=\dot{Q}C_{out}$), resulting in $U_{systematic}=\sqrt{{\dot{Q}}^{2}U_{C_{out}}^{2}+C_{out}^{2}U_{\dot{Q}}^{2}}$ (where $U_{C_{out}}$ and $U_{\dot{Q}}$ are the instrument uncertainties associated with the concentration and air flow described above). The total error was subsequently calculated as $U_{total}=\sqrt{U_{systematic}^{2}+U_{random}^{2}}$.

## References

[1] Aldea D., Atsuta Y., Kokalari B., Schaffner S.F., Prasasya R.D., Aharoni A., Dingwall H.L., Warder B., Kamberov Y.G. (2021). Repeated mutation of a developmental enhancer contributed to human thermoregulatory evolution. Proc. Natl. Acad. Sci. USA.

[2] Kuno Y. (1956).

[3] Havenith G., Richards M.G., Wang X., Bröde P., Candas V., den Hartog E., Holmér I., Kuklane K., Meinander H., Nocker W. (2008). Apparent latent heat of evaporation from clothing: attenuation and “heat pipe” effects. J. Appl. Physiol..

[4] Havenith G., Bröde P., den Hartog E., Kuklane K., Holmer I., Rossi R.M., Richards M., Farnworth B., Wang X. (2013). Evaporative cooling: effective latent heat of evaporation in relation to evaporation distance from the skin. J. Appl. Physiol..

[5] Rykaczewski K. (2020). Rational design of sun and wind shaded evaporative cooling vests for enhanced personal cooling in hot and dry climates. Appl. Therm. Eng..

[6] Ebi K.L., Capon A., Berry P., Broderick C., de Dear R., Havenith G., Honda Y., Kovats R.S., Ma W., Malik A. (2021). Hot weather and heat extremes: health risks. Lancet.

[7] Ebi K.L., Vanos J., Baldwin J.W., Bell J.E., Hondula D.M., Errett N.A., Hayes K., Reid C.E., Saha S., Spector J., Berry P. (2021). Extreme weather and climate change: population health and health system implications. Annu. Rev. Public Health.

[8] Jay O., Capon A., Berry P., Broderick C., de Dear R., Havenith G., Honda Y., Kovats R.S., Ma W., Malik A. (2021). Reducing the health effects of hot weather and heat extremes: from personal cooling strategies to green cities. Lancet.

[9] Derby B. (2010). Inkjet printing of functional and structural materials: fluid property requirements, feature stability, and resolution. Annu. Rev. Mater. Res..

[10] Brutin D., Starov V. (2018). Recent advances in droplet wetting and evaporation. Chem. Soc. Rev..

[11] Plawsky J.L., Fedorov A.G., Garimella S.V., Ma H.B., Maroo S.C., Chen L., Nam Y. (2014). Nano-and microstructures for thin-film evaporation—A review. Nanoscale Microscale Thermophys. Eng..

[12] Zang D., Tarafdar S., Tarasevich Y.Y., Dutta Choudhury M., Dutta T. (2019). Evaporation of a Droplet: From physics to applications. Phys. Rep..

[13] Li X., Guo W., Hsu P.C. (2024). Personal Thermoregulation by Moisture-Engineered Materials. Adv. Mater..

[14] Lei M., Li Y., Liu Y., Ma Y., Cheng L., Hu Y. (2020). Effect of weaving structures on the water wicking–evaporating behavior of woven fabrics. Polymers.

[15] Peng Y., Li W., Liu B., Jin W., Schaadt J., Tang J., Zhou G., Wang G., Zhou J., Zhang C. (2021). Integrated cooling (i-Cool) textile of heat conduction and sweat transportation for personal perspiration management. Nat. Commun..

[16] Raccuglia M., Heyde C., Lloyd A., Hodder S., Havenith G. (2018). Spatial and temporal migration of sweat: from skin to clothing. Eur. J. Appl. Physiol..

[17] Morris, N. B., Kjellstrom, T., Ioannou, L. G., Gao, C., Morabito, M., Messeri, A., Levi, M., Baldasseroni, A., Havenith, G., and Foster, J. (2019). Heat Shield Project D3. 6: Report on Solutions to Mitigate Heat Stress for Workers of the Agricultural Sector.

[18] Ioannou L.G., Foster J., Morris N.B., Piil J.F., Havenith G., Mekjavic I.B., Kenny G.P., Nybo L., Flouris A.D. (2022). Occupational heat strain in outdoor workers: A comprehensive review and meta-analysis. Temperature.

[19] Ravanelli N.M., Hodder S.G., Havenith G., Jay O. (2015). Heart rate and body temperature responses to extreme heat and humidity with and without electric fans. J. Am. Med. Assoc..

[20] Gagnon D., Romero S.A., Cramer M.N., Jay O., Crandall C.G. (2016). Cardiac and thermal strain of elderly adults exposed to extreme heat and humidity with and without electric fan use. J. Am. Med. Assoc..

[21] Filingeri D., Havenith G. (2015). Human skin wetness perception: psychophysical and neurophysiological bases. Temperature.

[22] Filingeri D. (2016). Neurophysiology of skin thermal sensations. Compr. Physiol..

[23] Lolla V.Y., Shukla P., Jones S.D., Boreyko J.B. (2020). Evaporation-Induced Clogging of an Artificial Sweat Duct. ACS Appl. Mater. Interfaces.

[24] Keshavarzi F., Knudsen N.Ø., Komjani N.M., Ebbesen M.F., Brewer J.R., Jafarzadeh S., Thormann E. (2022). Enhancing the sweat resistance of sunscreens. Skin Res. Technol..

[25] Davis N., Heikenfeld J., Milla C., Javey A. (2024). The challenges and promise of sweat sensing. Nat. Biotechnol..

[26] Zhang B., Li J., Zhou J., Chow L., Zhao G., Huang Y., Ma Z., Zhang Q., Yang Y., Yiu C.K. (2024). A three-dimensional liquid diode for soft, integrated permeable electronics. Nature.

[27] Yang H., Ding H., Wei W., Li X., Duan X., Zhuang C., Liu W., Chen S., Wang X. (2024). Skin-interfaced microfluidic sweat collection devices for personalized hydration management through thermal feedback. Lab Chip.

[28] Liu Y., Li X., Yang H., Zhang P., Wang P., Sun Y., Yang F., Liu W., Li Y., Tian Y. (2023). Skin-interfaced superhydrophobic insensible sweat sensors for evaluating body thermoregulation and skin barrier functions. ACS Nano.

[29] Baker L.B. (2019). Physiology of sweat gland function: The roles of sweating and sweat composition in human health. Temperature.

[30] Taylor N.A., Machado-Moreira C.A. (2013). Regional variations in transepidermal water loss, eccrine sweat gland density, sweat secretion rates and electrolyte composition in resting and exercising humans. Extrem. Physiol. Med..

[31] Smith C.J., Havenith G. (2011). Body mapping of sweating patterns in male athletes in mild exercise-induced hyperthermia. Eur. J. Appl. Physiol..

[32] Gerrett N., Griggs K., Redortier B., Voelcker T., Kondo N., Havenith G. (2018). Sweat from gland to skin surface: production, transport, and skin absorption. J. Appl. Physiol..

[33] Coull N.A., West A.M., Hodder S.G., Wheeler P., Havenith G. (2021). Body mapping of regional sweat distribution in young and older males. Eur. J. Appl. Physiol..

[34] Sonner Z., Wilder E., Heikenfeld J., Kasting G., Beyette F., Swaile D., Sherman F., Joyce J., Hagen J., Kelley-Loughnane N., Naik R. (2015). The microfluidics of the eccrine sweat gland, including biomarker partitioning, transport, and biosensing implications. Biomicrofluidics.

[35] Candas V., Libert J.P., Vogt J.J. (1979). Human skin wettedness and evaporative efficiency of sweating. J. Appl. Physiol..

[36] Candas V., Libert J.P., Vogt J.J. (1979). Influence of air velocity and heat acclimation on human skin wettedness and sweating efficiency. J. Appl. Physiol..

[37] Tam H.-S., Darling R.C., Downey J.A., Cheh H.Y. (1976). Relationship between evaporation rate of sweat and mean sweating rate. J. Appl. Physiol..

[38] Parsons K. (2014).

[39] Wissler E.H. (2018).

[40] PARK S.J., TAMURA T. (1992). Distribution of evaporation rate on human body surface. Ann. Physiol. Anthropol..

[41] Holmér I. (2004). Thermal manikin history and applications. Eur. J. Appl. Physiol..

[42] Psikuta A., Allegrini J., Koelblen B., Bogdan A., Annaheim S., Martínez N., Derome D., Carmeliet J., Rossi R.M. (2017). Thermal manikins controlled by human thermoregulation models for energy efficiency and thermal comfort research – A review. Renew. Sustain. Energy Rev..

[43] Gholamreza F., Su Y., Li R., Nadaraja A.V., Gathercole R., Li R., Dolez P.I., Golovin K., Rossi R.M., Annaheim S., Milani A.S. (2022). Modeling and Prediction of Thermophysiological Comfort Properties of a Single Layer Fabric System Using Single Sector Sweating Torso. Materials.

[44] Wang F. (2011). Physiological Model Controlled Sweating Thermal Manikin: Can it replace human subjects?. J. Ergonom..

[45] Gao H., Shawn Deaton A., Barker R. (2022). A new test method for evaluating the evaporative cooling efficiency of fabrics using a dynamic sweating hot plate. Meas. Sci. Technol..

[46] Huang J. (2006). Sweating guarded hot plate test method. Polym. Test..

[47] Kim H.s., Michielsen S., DenHartog E. (2020). New wicking measurement system to mimic human sweating phenomena with continuous microfluidic flow. J. Mater. Sci..

[48] Rossi R.M. (2017).

[49] Guan M., Annaheim S., Camenzind M., Li J., Mandal S., Psikuta A., Rossi R.M. (2019). Moisture transfer of the clothing–human body system during continuous sweating under radiant heat. Textil. Res. J..

[50] Rabost-Garcia G., Farré-Lladós J., Casals-Terré J. (2021). Recent Impact of Microfluidics on Skin Models for Perspiration Simulation. Membranes.

[51] Koelblen B., Psikuta A., Bogdan A., Annaheim S., Rossi R.M. (2017). Comparison of fabric skins for the simulation of sweating on thermal manikins. Int. J. Biometeorol..

[52] Incropera F.P., DeWitt D.P., Bergman T.L., Lavine A.S. (2007). Fundamentals of Heat and Mass Transfer.

[53] Meade R.D., Louie J.C., Poirier M.P., McGinn R., Fujii N., Kenny G.P. (2016). Exploring the mechanisms underpinning sweating: the development of a specialized ventilated capsule for use with intradermal microdialysis. Physiol. Rep..

[54] Rutherford M.M., Akerman A.P., Notley S.R., Meade R.D., Schmidt M.D., Kenny G.P. (2021). Regional variation in the reliability of sweat rate measured via the ventilated capsule technique during passive heating. Exp. Physiol..

[55] Lamke L.-O. (1970). An instrument for estimating evaporation from small skin surfaces. Scand. J. Plast. Reconstr. Surg..

[56] Nicolaidis S., Sivadjian J. (1972). High-frequency pulsatile discharge of human sweat glands: myoepithelial mechanism. J. Appl. Physiol..

[57] Jonathan E. (2008). In vivo sweat film layer thickness measured with Fourier-domain optical coherence tomography (FD-OCT). Opt. Lasers Eng..

[58] Kato K., Al-Sobaihi S., Al-Busani H., Nishizawa A., Ohmi M., Yokozeki H., Namiki T. (2021). Analysis of sweating by optical coherence tomography in patients with palmoplantar hyperhidrosis. J. Dermatol..

[59] Ohmi M., Nohara K., Ueda Y., Fuji T., Haruna M. (2005). Dynamic observation of sweat glands of human finger tip using all-optical-fiber high-speed optical coherence tomography. Jpn. J. Appl. Phys..

[60] Ohmi M., Tanigawa M., Wada Y., Haruna M. (2012). Dynamic analysis for mental sweating of a group of eccrine sweat glands on a human fingertip by optical coherence tomography. Skin Res. Technol..

[61] Ohmi M., Wada Y. (2016). 2016 38th Annual International Conference of the IEEE Engineering in Medicine and Biology Society (EMBC).

[62] Sagaidachnyi A., Mayskov D., Fomin A., Zaletov I., Skripal A. (2022). Separate extraction of human eccrine sweat gland activity and peripheral hemodynamics from high-and low-quality thermal imaging data. J. Therm. Biol..

[63] Mayskov D.I., Fomin A.V., Volkov I.U., Zaletov I.S., Skripal A.V., Sagaidachnyi A.A. (2022). Optical Technologies for Biology and Medicine.

[64] Drexelius A., Fehr D., Vescoli V., Heikenfeld J., Bonmarin M. (2022). A simple non-contact optical method to quantify in-vivo sweat gland activity and pulsation. IEEE Trans. Biomed. Eng..

[65] Krzywicki A.T., Berntson G.G., O’Kane B.L. (2014). A non-contact technique for measuring eccrine sweat gland activity using passive thermal imaging. Int. J. Psychophysiol..

[66] Koroteeva E.Y., Bashkatov A.A. (2021). Thermal signatures of liquid droplets on a skin induced by emotional sweating. Quant. InfraRed Thermogr. J..

[67] Chandramohan A., Weibel J.A., Garimella S.V. (2017). Spatiotemporal infrared measurement of interface temperatures during water droplet evaporation on a nonwetting substrate. Appl. Phys. Lett..

[68] Gleason K., Voota H., Putnam S.A. (2016). Steady-state droplet evaporation: Contact angle influence on the evaporation efficiency. Int. J. Heat Mass Transf..

[69] Hernández, M.A.G., López, A.I.M., Jarzabek, A.A., Perales, J.M.P., Wu, Y., and Xiaoxiao, S. (2013). Design methodology for a quick and low-cost wind tunnel. Wind tunnel designs and their diverse engineering applications, 1.

[70] Bergman T.L., Lavine A.S., Incropera F.P., Dewitt D.P. (2011).

[71] Randall W.C. (1946). Sweat gland activity and changing patterns of sweat secretion on the skin surface. Am. J. Physiol..

[72] Albert R.E., Palmes E.D. (1951). Evaporative rate patterns from small skin areas as measured by an infrared gas analyzer. J. Appl. Physiol..

[73] Machado-Moreira C.A., Taylor N.A.S. (2017). Thermogenic and psychogenic recruitment of human eccrine sweat glands: Variations between glabrous and non-glabrous skin surfaces. J. Therm. Biol..

[74] McGregor I.A. (1952). The sweating reactions of the forehead. J. Physiol..

[75] Nilsson A.L., Nilsson G.E., Öberg P.A. (1982). On periodic sweating from the human skin during rest and exercise. Acta Physiol. Scand..

[76] Nishiyama T., Sugenoya J., Matsumoto T., Iwase S., Mano T. (2001). Irregular activation of individual sweat glands in human sole observed by a videomicroscopy. Auton. Neurosci..

[77] Ogawa T., Sugenoya J. (1993). Pulsatile sweating and sympathetic sudomotor activity. Jpn. J. Physiol..

[78] Schwarck J.B., Burdon C.A., Taylor E.A., Peoples G.E., Machado-Moreira C.A., Taylor N.A.S. (2019). Thermogenic and psychogenic sweating in humans: Identifying eccrine glandular recruitment patterns from glabrous and non-glabrous skin surfaces. J. Therm. Biol..

[79] Morin M., Ruzgas T., Svedenhag P., Anderson C.D., Ollmar S., Engblom J., Björklund S. (2020). Skin hydration dynamics investigated by electrical impedance techniques in vivo and in vitro. Sci. Rep..

[80] Quéré D. (2008). Wetting and Roughness. Annu. Rev. Mater. Res..

[81] Bromberg L., Liu X., Wang I., Smith S., Schwicker K., Eller Z., German G.K. (2017). Control of human skin wettability using the pH of anionic surfactant solution treatments. Colloids Surf. B Biointerfaces.

[82] Ginn M.E., Noyes C.M., Jungermann E. (1968). The contact angle of water on viable human skin. J. Colloid Interface Sci..

[83] Eudier F., Savary G., Grisel M., Picard C. (2019). Skin surface physico-chemistry: Characteristics, methods of measurement, influencing factors and future developments. Adv. Colloid Interface Sci..

[84] Akkus Y., Çetin B., Dursunkaya Z. (2019). An iterative solution approach to coupled heat and mass transfer in a steadily fed evaporating water droplet. J. Heat Transfer.

[85] Akkus Y., Çetin B., Dursunkaya Z. (2020). A theoretical framework for comprehensive modeling of steadily fed evaporating droplets and the validity of common assumptions. Int. J. Therm. Sci..

[86] Bullard R.W. (1971). Studies on human sweat gland duct filling and skin hydration. J. Physiol..

[87] MacPherson R.K., Newling P.S.B. (1954). JOURNAL OF PHYSIOLOGY-LONDON.

[88] Hancock W., Whitehouse A.G.R., Haldane J.S. (1929). The loss of water and salts through the skin, and the corresponding physiological adjustments. Proc. R. Soc. Lond. - Ser. B Contain. Pap. a Biol. Character.

[89] ASTM (1985).

[90] Morris N.B., Cramer M.N., Hodder S.G., Havenith G., Jay O. (2013). A comparison between the technical absorbent and ventilated capsule methods for measuring local sweat rate. J. Appl. Physiol..

[91] Rasband W.S. (2011). imagej. http://imagej.nih.gov/ij/.

