# Supplementary material for: Simultaneous imaging of multi-pore sweat dynamics and evaporation rate measurement using wind tunnel ventilated capsule with infrared window
Source: iScience. 2024 Jun 17;27(7):110304. doi: 10.1016/j.isci.2024.110304 (PMC11261446; doi:10.1016/j.isci.2024.110304)
Supplement: Document S1. Figures S1–S7 and Table S1 [file mmc1.pdf]

iScience, Volume *27*

Supplemental information

Simultaneous imaging of multi-pore sweat dynamics and evaporation rate measurement using wind tunnel ventilated capsule with infrared window

Ankush K. Jaiswal, Cibin T. Jose, Rajesh Ramesh, Vinay K. Nanani, Kambiz Sadeghi, Ankit Joshi, Krishna Kompally, Gokul Pathikonda, Heather N. Emady, Bhaumik Bheda, Stavros A. Kavouras, and Konrad Rykaczewski

Wind-tunnel ventilated capsule geometry and assembly

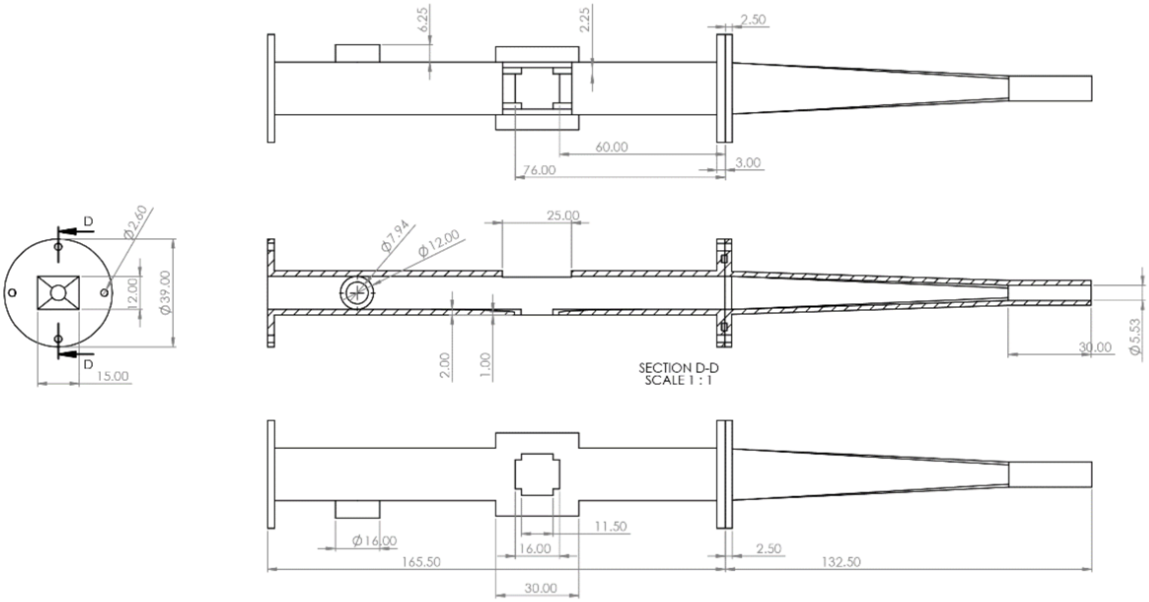

Figure S1. The technical drawing of the wind tunnel ventilated capsule (units: mm), related to STAR Methods. Step-by-step assembly images are shown elsewhere.[S1]

Ventilated capsule water absorption and air leak tests, and humidity probe calibration

Table S1. Mass of printer parts coated with epoxy before and after 24h soaking in water related to water absorption testing described in the STAR Methods.

	Sample 1	Sample 2
Mass: before (±0.001 g)	3.139	9.924
Mass: after (±0.001 g)	3.145	10.042
Change in mass (±0.001 g)	0.006	0.118
Percentage change (%)	0.2	1.2

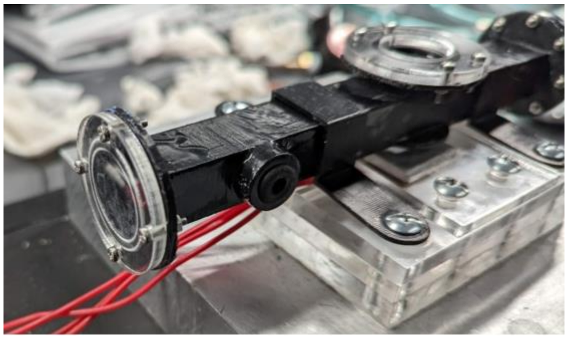

Figure S2. Image of the capsule setup used in air leak tests related to STAR Methods. The cap installed on the end of the ventilated capsule for the air leak tests is shown.

The heated square water film setup

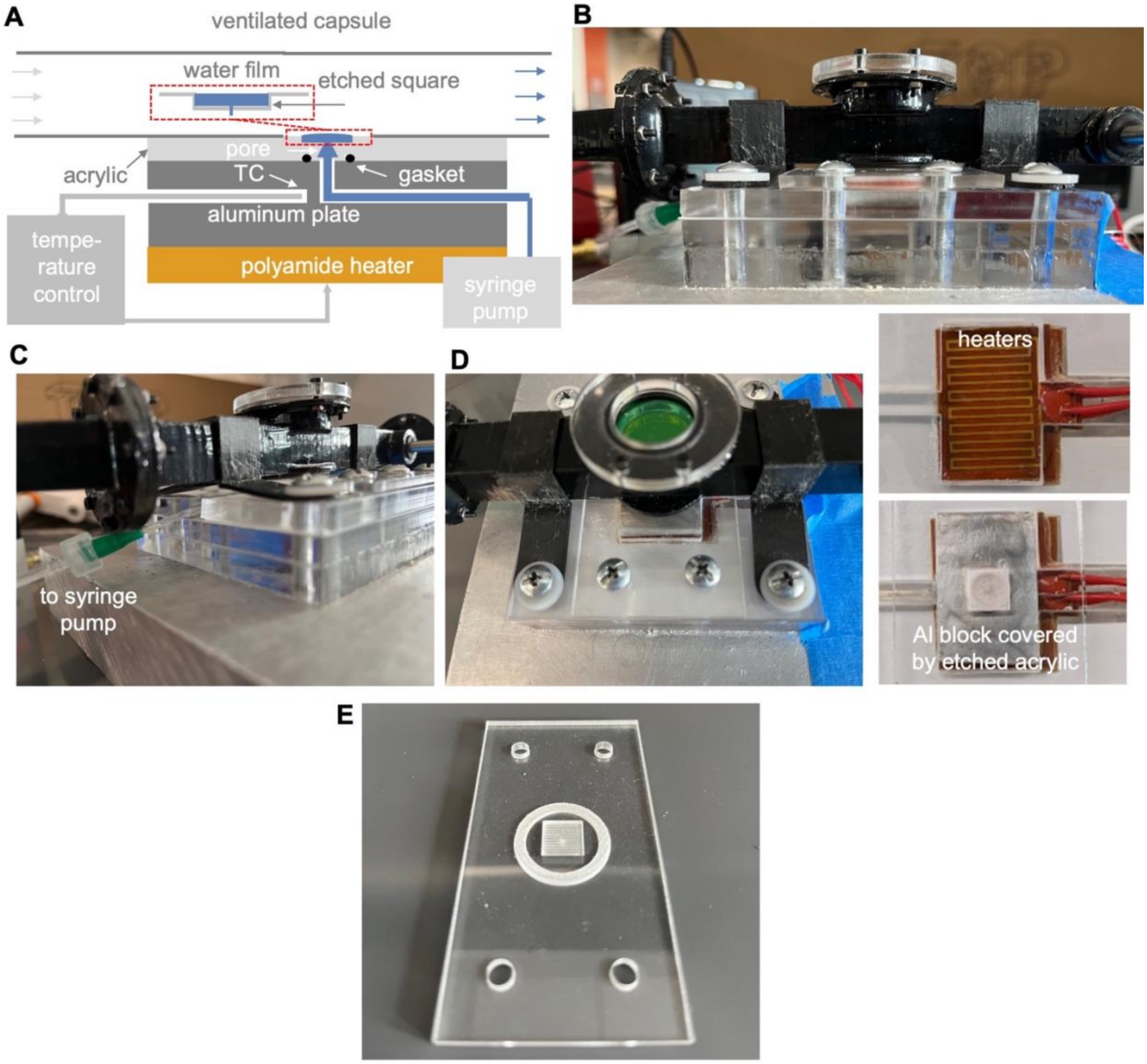

Figure S3. Images of the heated, square water film "artificial sweating" setup related to STAR Methods. A.) Schematic diagram, **B-D.)** images of the entire assembly with the capsule as well as images of partial assembly revealing the heaters and metal block, **E.)** example image of the double-side etched acrylic part (o-ring groove in on bottom, square film is on top, and the acrylic is pierced through with 0.1 mm hole in center of the square).

Multiphysics simulation formulation

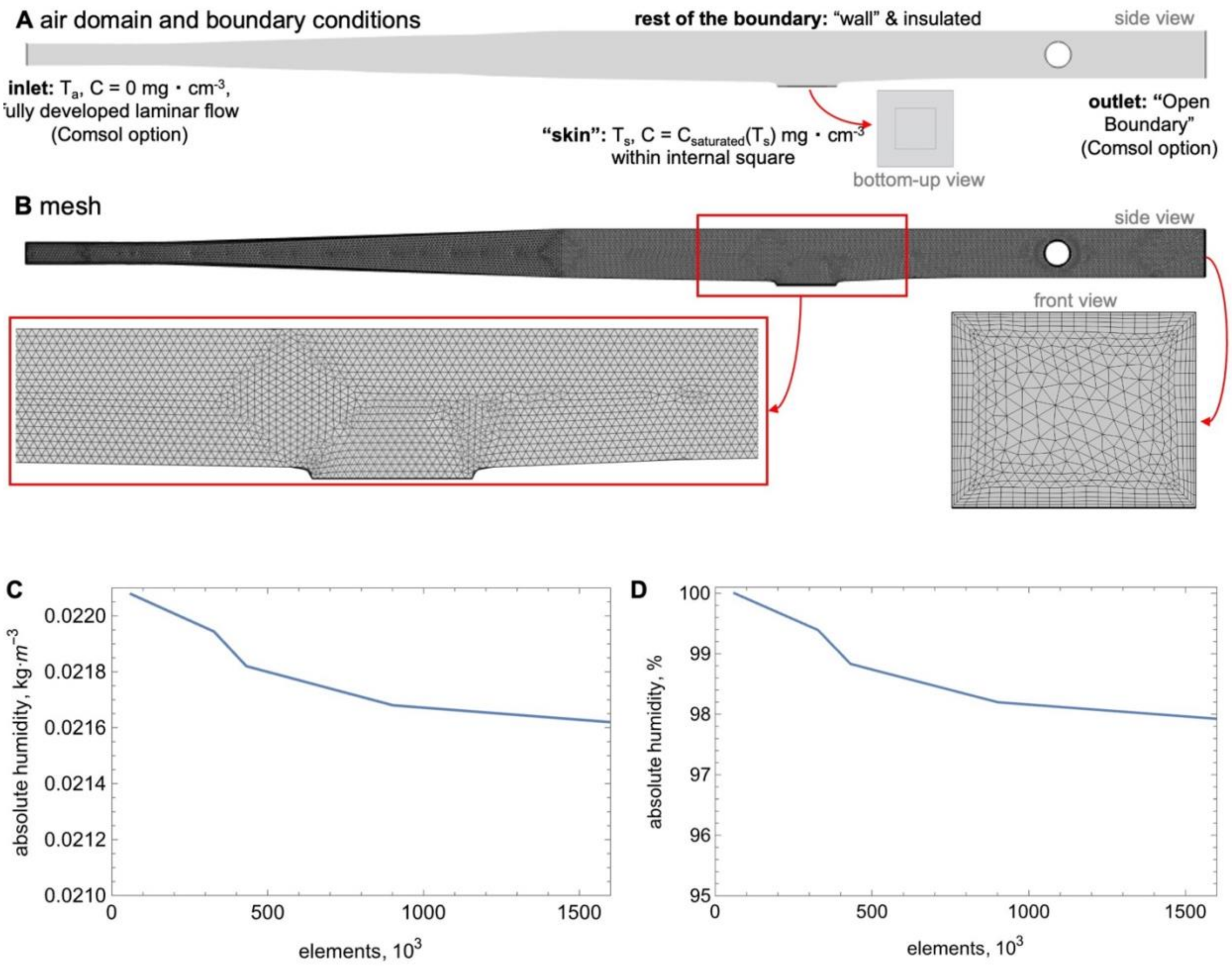

Figure S4. The multiphysics simulation formulation and mesh refinement study related to STAR Methods. A.) Schematic of the air domain boundary conditions, **B.)** Schematics of the final model mesh, **and C.)** and **D.)** the mesh refinement study illustrating 0.5% change in outlet humidity with increase of mesh elements from 900,000 to 1600,000 (finer to extremely fine settings).

Flow in 6° half angle diffuser and sudden expansion sections

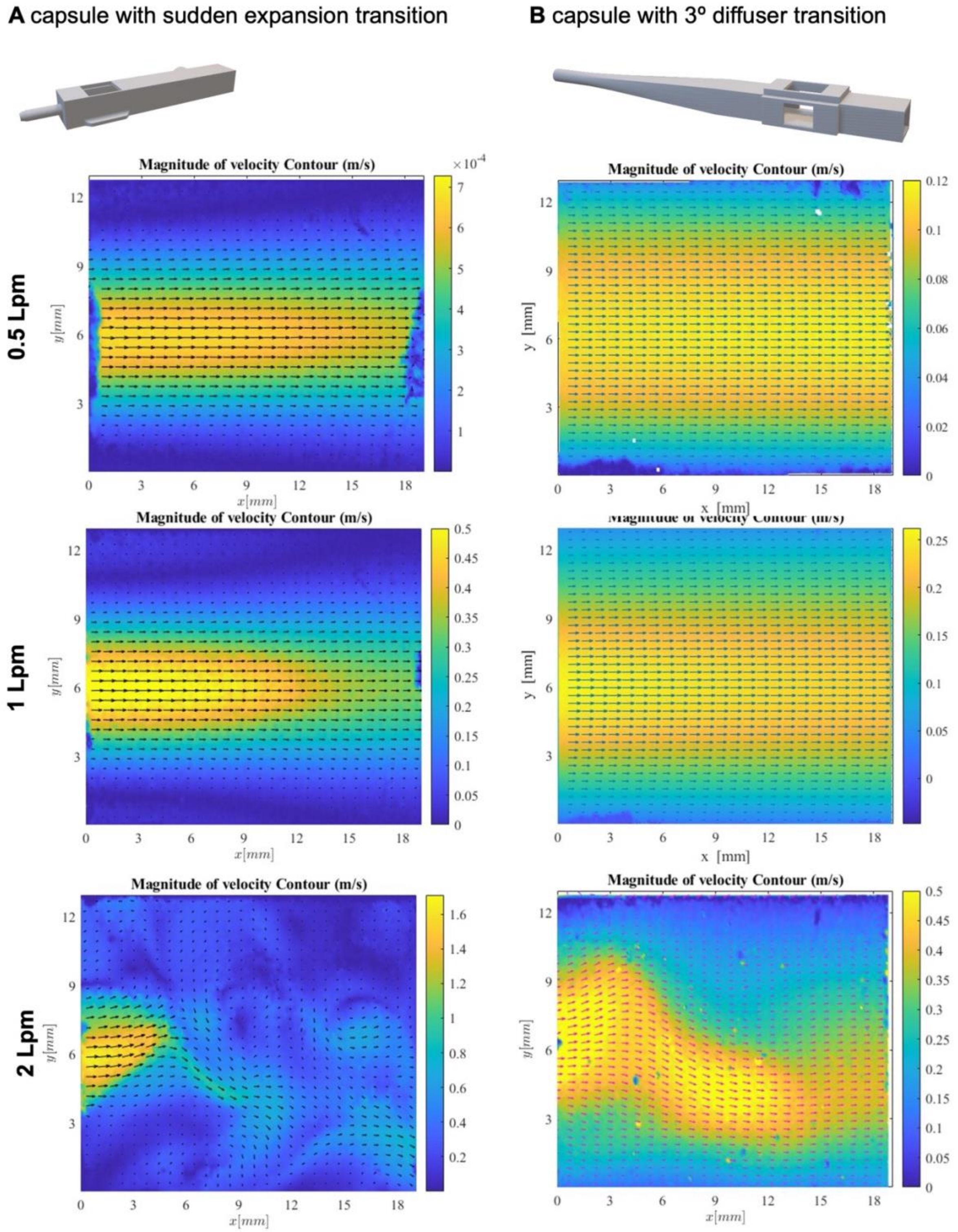

Figure S5. Flow in 6° half angle diffuser and sudden expansion sections related to STAR Methods. A.) PIV visualized flow in a ventilated capsule with a sudden tube to rectangular section transition and **B.)** with 3° half angle diffuser.

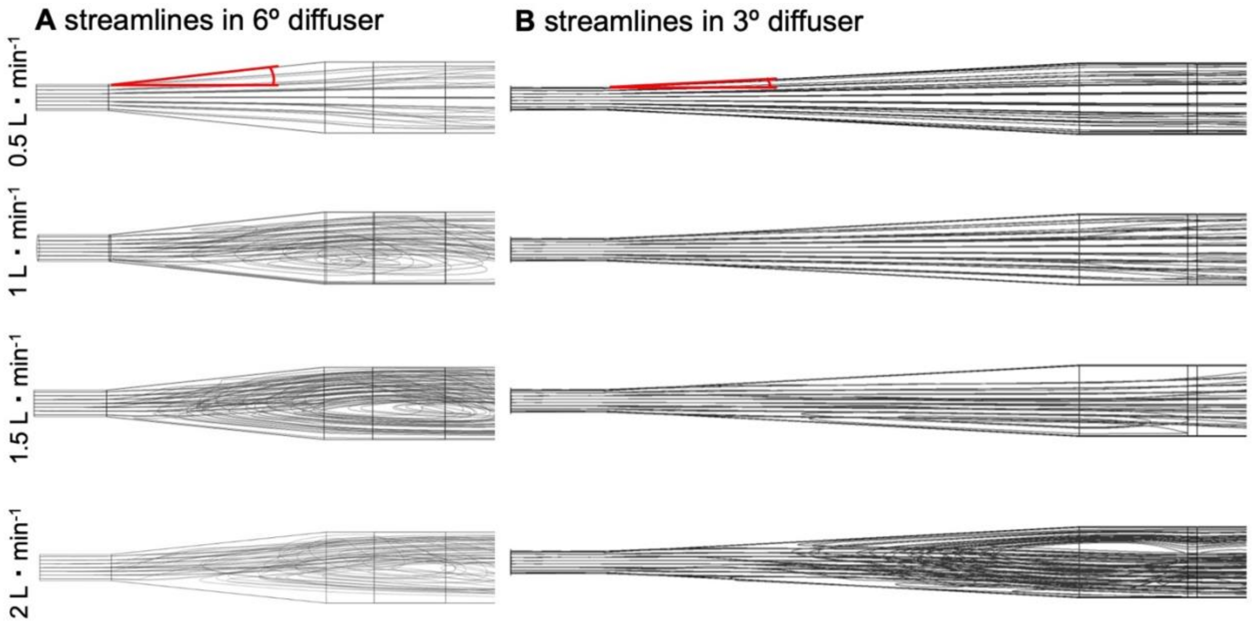

Figure S6. Simulated flow in a ventilated capsule related to STAR Methods. A.) flow within capsule with a 6° half angle diffuser and **B.)** with a 3° half angle diffuser for varied volumetric air flow rates.

The experimental setup for human subject experimentation

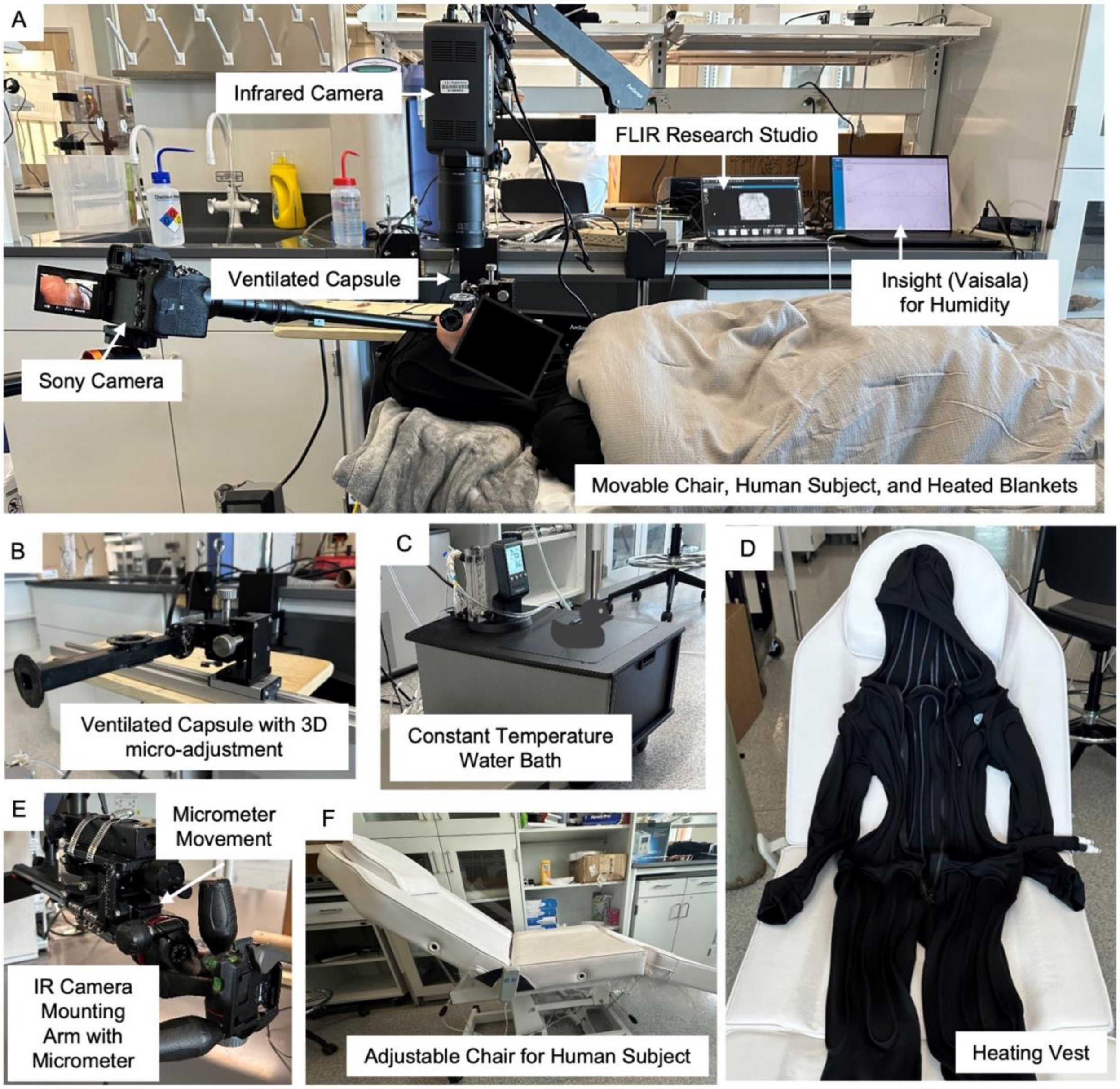

Figure S7. Images of the experimental setup for human subject experimentation related to STAR Methods. A.) Experimental setup for analyzing microscale features of human sweat subject to heating **B.)** ventilated capsule and arrangement for moving is in micrometer values in all 3-dimensions **C.)** constant temperature water bath for heating **D.)** full body heating vest connected to bath E. MWIR camera mount with provision for adjusting its movement in micrometer and F. adjustable chair with medical grade sheet and additional heating blankets.

References

[S1]. Ramesh, R. (2023). Ventilated capsule for sweat evaporation rate measurement. Master's Thesis. Arizona State University.
